# Contrasting Metabolisms in Green and White Leaf Sectors of Variegated *Pelargonium zonale*—An Integrative Transcriptomic and Metabolomic Study

**DOI:** 10.3390/ijms24065288

**Published:** 2023-03-09

**Authors:** Dejana Milić, Ana Pantelić, Bojana Banović Đeri, Jelena Samardžić, Marija Vidović

**Affiliations:** University of Belgrade, Institute of Molecular Genetics and Genetic Engineering, Laboratory for Plant Molecular Biology, Vojvode Stepe 444a, 11042 Belgrade, Serbia

**Keywords:** de novo transcriptomic assembly, metabolomics, sink-source leaf tissue, variegated leaves, *Pelargonium zonale*

## Abstract

The photosynthetically active green leaf (GL) and non-active white leaf (WL) tissues of variegated *Pelargonium zonale* provide an excellent model system for studying processes associated with photosynthesis and sink-source interactions, enabling the same microenvironmental conditions. By combining differential transcriptomics and metabolomics, we identified the main differences between these two metabolically contrasting tissues. Genes related to photosynthesis and associated pigments, the Calvin–Benson cycle, fermentation, and glycolysis were strongly repressed in WL. On the other hand, genes related to nitrogen and protein metabolism, defence, cytoskeletal components (motor proteins), cell division, DNA replication, repair and recombination, chromatin remodelling, and histone modifications were upregulated in WL. A content of soluble sugars, TCA intermediates, ascorbate, and hydroxybenzoic acids was lower, while the concentration of free amino acids (AAs), hydroxycinnamic acids, and several quercetin and kaempferol glycosides was higher in WL than in GL. Therefore, WL presents a carbon sink and depends on photosynthetic and energy-generating processes in GL. Furthermore, the upregulated nitrogen metabolism in WL compensates for the insufficient energy from carbon metabolism by providing alternative respiratory substrates. At the same time, WL serves as nitrogen storage. Overall, our study provides a new genetic data resource for the use of this excellent model system and for ornamental pelargonium breeding and contributes to uncovering molecular mechanisms underlying variegation and its adaptive ecological value.

## 1. Introduction

Foliar variegation is a common phenotype, observed in many angiosperms. Variegated leaves typically contain green and white or yellowish sectors. The variegated *Pelargonium zonale* cv. Frank Headley is a stable periclinal chimaera, with a green sector in the middle of the leaf blade and a white leaf margin. The photosynthetically active cells in the green leaf (GL) sectors contain physiologically developed chloroplasts with well-organised thylakoids and starch grains, while the non-photosynthetic cells in the white leaf (WL) sectors lack functional chloroplasts and peroxisomes [1]. Previous studies used the green-white leaf variegation of *P. zonale* to investigate the inheritance of plastid and mitochondrial genomes [2,3,4,5,6].

Impaired chloroplast development is considered as a hallmark of albino phenotype in variegated leaves. Proper chloroplast differentiation is a complex process, coordinated with peroxisomes, mitochondria, and cytoskeleton, and requires regulation and cooperation between nuclear and chloroplast genes (involving retrograde signalling) [7]. Cytoskeletal components are important for the development of stromules, mobile and transitory chloroplast extensions involved in the interorganellar transport of proteins and metabolites [8]. 

To the best of our knowledge, the molecular basis for the mutation responsible for the development of white leaf sectors in variegated *Pelargonium* species has not yet been elucidated. The molecular mechanisms responsible for the variegated phenotype have been studied in several species (including Arabidopsis), but no single and unique cause of variegation has been found [9,10,11]. For example, the variegated Arabidopsis mutant, *yellow variegated2* (*var2*), lacks FtsH2, a thylakoid-bound protease involved in the degradation of the oxidised D1 subunit of the photosystem II (PSII) [12,13], and functions as a scaffolding protein during thylakoid development [14]. This finally leads to increased PSII photoinhibition and oxidative stress, which in turn results in chloroplast degradation. On the other hand, variegation in the Arabidopsis mutant *immutans* (*im*) is a consequence of the absence of the plastid terminal oxidase IM, which controls the activity of phytoene desaturase, involved in carotenoid biosynthesis [15]. In addition, this ubiquitous protein is homologous to the alternative oxidase (AOX) of the inner mitochondrial membranes. The absence of IM eventually leads to irregular organisation of the thylakoid lamellar structures and to an over-reduction of the plastoquinone pool, resulting in chlorophyll photooxidation in *immutans* WL [16]. Recently, it was suggested that suppression of the mitochondrial transcription termination factor (mTERF) alters chloroplast development in albino leaf sectors of the variegated *Ficus microcarpa* (cv. Milky Stripe Fig) [17]. On the other hand, the absence of a peroxisomal microtubule-associated protein—SCO3—causes the albino phenotype in variegated Arabidopsis *snowy cotyledon3* (*sco3-1*) mutants. SCO3 is suggested to alter actin fine structure and disrupt protein import into chloroplasts through stromules, leading to impaired chloroplast development [18]. Furthermore, the variegation can be attributed to transposons [9,19].

Natural variegation of *P. zonale* leaves proved to be an excellent model for comparing photosynthetically active and photosynthetically non-active tissues in terms of H_2_O_2_ production within the same leaf providing the same microenvironmental conditions [20,21]. In green leaves, reactive oxygen species (ROS) are mainly produced by photorespiration and the photosynthetic electron transport chain (ETC), even under physiological growth conditions [22]. Since WL lacks functional chloroplasts and peroxisomes, the main sources of ROS in WL are the electron transport chain in the mitochondria and apoplast [23,24]. The respiration rate in WL of variegated *P. zonale* is significantly lower than in GL [25]. We have shown that *P. zonale* WL has constitutively higher activities of enzymes involved in the ascorbate–glutathione cycle (Asc-GSH) and Cu/Zn superoxide dismutase (SOD) compared to GL [20]. Furthermore, higher cytosolic ascorbate (Asc) and total cellular glutathione (GSH) levels were detected in WL than in GL. On the other hand, higher total Asc content and higher activities of catalase (CAT) and thylakoid ascorbate peroxidase (tAPX) were observed in GL [20].

Secondly, variegated pelargonium is a suitable model system to study source-sink interactions within the same leaf with respect to sugars, polyphenols, and amino acids (AAs) [1,26,27]. Autotrophic leaf tissue has been shown to contain higher levels of soluble sugars, while heterotrophic leaf tissue has higher levels of nitrogenous compounds, especially free AAs.

Moreover, variegated leaf phenotypes often increase the commercial value of ornamental plants [19,28]. Pelargoniums are popular ornamental garden plants with significant economic impact. In 2021, the value of the flower market at the world’s largest flower exchange, Royal Flora Holland, reached 5.6 billion euros [29]. Pelargonium is the most important garden plant traded in Europe, and Germany, the United Kingdom, France, and Belgium are the leading importing countries [30]. The total European trade of pelargonium amounted to 13 million euros in 2017 at Royal Flora Holland, with a market share of 8.2% for garden plants [31].

The aim of our study was to identify the main metabolic differences between GL and WL of the variegated *P. zonale* plant. This ornamental plant species was chosen because it has proven to be an exceptional model system to study the mechanisms underlying the regulation of chlorophyll biosynthesis, chloroplast structure, function, and development, and the influence of sink leaf tissue on photosynthesis. To achieve our goal, a systems biology approach combining transcriptomics and metabolomics analyses was adopted. For the first time, we performed a de novo transcriptome analysis of *P. zonale* and analysed the differentially expressed genes (DEGs) in GL and WL. The differential gene expression analysis of GL and WL was correlated with metabolic pathways, metal content, and enzyme activities.

## 2. Results

The aim of this study was to reveal the main transcriptomic and metabolomic differences between photosynthetically active green leaf tissue (GL) and photosynthetically inactive white leaf tissue (WL) of *P. zonale*. In this study, the first transcriptomic database of *P. zonale* is presented.

### 2.1. De Novo Transcriptome Assembly of P. zonale GL and WL

Highly purified cDNA libraries from GL and WL (presented in Vidovic and Ćuković [32]) were sequenced and filtered, resulting in 41680883 (G2 sample, with Q30 = 94%) and 45337775 (W3 sample, with Q30 = 94.1%) clean reads, that were used for P. zonale de novo transcriptome assembly. After transcriptome analysis, 139811 transcripts with 139575 unigenes with a mean sequence length of 1220 nt, and an N50 value of 1874 nt were obtained (Supplementary Table S1).

### 2.2. Gene Functional Analysis

After *P. zonale* de novo transcriptome interpretation, 85,374 unigenes (61.17%) were annotated in at least one of the seven common databases. Of the unigenes obtained, 59.33% and 44.01% were annotated in the NCBI Non-Redundant Protein (NR) and Nucleotide Sequences database (NT), respectively, 23.61% in the Protein Families database (Pfam), 19.91% in the EuKaryotic Orthologous Groups database (KOG), 46.31% in the Swiss-Prot database, 20.86% in the Kyoto Encyclopaedia of Genes and Genome (KEGG), and 14.89% in the Gene Ontology database (GO), respectively. The E-value distribution in *P. zonale* leaf tissue showed that 32.0% of the annotated unigenes had E-values ranging from 0 to 1 × 10^−100^, followed by 16% with E-values ranging from 1 × 10^−100^ to 1 × 10^−60^ (Supplementary Figure S1A). Nearly 80.6% of the unigenes had a strong similarity of more than 80%, while 6.7% of the unigenes had a similarity value between 40% and 80% (Supplementary Figure S1B). The species distribution analysis showed that 9.6% of the annotated sequences had the best matches with sequences of *Quercus suber* (Supplementary Figure S1C). Besides *Q. suber,* the highest similarity with *P. zonale* transcripts was found in *Vitis vinifera* (8.2%)*, Juglans regia* (5.3%), *Hevea brasiliensis* (4.2%), and *Theobroma cacao* (3.6%).

In the Gene Ontology (GO) analysis, 20,778 unigenes were assigned to three main categories GO (Supplementary Figure S2). Within the category of biological processes (BP), most unigenes were assigned to cellular processes (36.6%), organic substance metabolic process (15.8%), regulation of biological processes (10.3%), primary metabolic processes (7.4%), and nitrogen compound metabolic process (7.1%; Supplementary Figure S2A). In addition, unigenes related to the binding of cyclic compounds, small molecules, carbohydrate derivatives, and ions contributed with 58.9%, while hydrolases and transferases accounted for 14.6% of the molecular function (MF) category (Supplementary Figure S2B). Within the cellular component (CC) category, 55.2% of the unigenes were related to membrane-bound proteins (organellar membranes and plasma membranes), 21.1% to organellar soluble proteins, 2% to the nucleus, 1.8% to the endoplasmic reticulum, 1.5% to thylakoids, and 1.3% to mitochondria (Supplementary Figure S2C).

For the KOG annotation, 27,798 unigenes were annotated for *P. zonale* transcriptome. These unigenes were classified into 25 categories (Supplementary Figure S3). Among them, “General function prediction” (15%), “Posttranslational modification, protein turnover, chaperones” (12%), “Signal transduction mechanisms” (8%), “Translation ribosomal structure and biogenesis” (6%), and “Intracellular trafficking, secretion, and vesicular transport” (6%) were the most represented KOG pathways (Supplementary Figure S3).

### 2.3. Quantification

For all six samples analysed (3 × GL and 3 × WL), more than 80% of the total reads were successfully mapped back to the assembled transcriptome, and FPKM (Fragments Per Kilobase of transcript sequence per Millions base pair sequenced) values were calculated (for biological replicates, the mean FPKM value was used). The FPKM distribution diagram was used to compare gene expression levels between GL and WL (Supplementary Figure S4A). The FPKM distribution showed that some unigenes were expressed at different levels in both tissues, while others were expressed exclusively in one tissue. The differences found in gene expression between GL and WL are discussed in more detail in the following sections.

We also tested the correlation between samples as an important indicator of the experiment reliability. The square of the Pearson correlation coefficient between W2 and W3; W1 and W2; and W1 and W3 was 0.93, 0.89, and 0.75 respectively, while it was 0.92, 0.88, and 0.88 between G2 and G3; G1 and G2; and G1 and G3, respectively, indicating greater similarities between GL than WL samples (Supplementary Figure S4B).

### 2.4. Differentially Expressed Genes of P. zonale GL and WL

**Figure 1:**
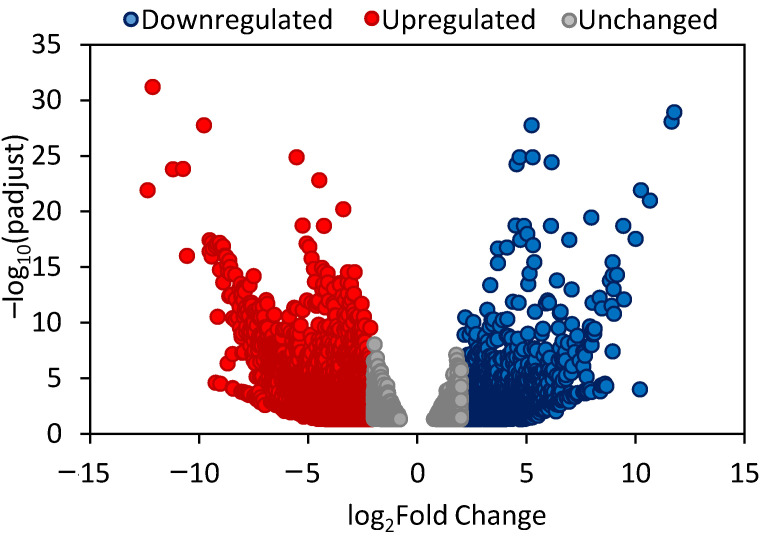
Identified differentially expressed gene clusters (DEGs) between *P. zonale* GL and WL. Volcano plots showing the expression level of each unigene. Limits defined by *p*-value ≤ 0.05 and abs (log2 fold change) ≥ 2. Red dots represent up-regulated genes in WL, blue dots represent down-regulated genes in WL, and grey dots represent statistically unchanged genes.

From the total of 139,575 gene clusters obtained in the transcriptome of *P. zonale* leaves, 8896 gene clusters were found to be statistically significant differentially expressed gene clusters (DEGs) between GL and WL using a more stringent threshold (*p* value < 0.05 and abs (log2 fold change) ≥ 2) (Supplementary Table S2). Among these, 5585 gene clusters were upregulated in WL and 3311 genes were upregulated in GL (Figure 1).

A clear clustering of genes that showed an opposite expression pattern between WL and GL was observed, with multiple gene clusters upregulated in WL and downregulated in GL and vice versa (Supplementary Figure S5).

To validate the determined DEGs, we selected three upregulated and three downregulated genes in WL (Supplementary Table S4) and performed qRT-PCR. The qRT-PCR results were strongly correlated with the RNAseq data (R^2^ = 0.91, Supplementary Figure S6). This confirms the high reliability of the obtained RNAseq data.

### 2.5. Functional Classification of DEGs in P. zonale Leaves

Enrichment analysis of DEGs allowed us to reveal several biological functions/pathways significantly associated with GL and WL. A total of 7294 DEGs between *P. zonale* GL and WL were assigned to three main categories GO (Supplementary Table S3). Results of GO enrichment showed that statistically significant differences (*P*adjust ≤ 0.05) between *P. zonale* GL and WL included 840 gene clusters assigned to: two different BP subcategories related to photosynthesis (18 DEG clusters) and transport (369 DEG clusters); four different CC subcategories related to cytoskeleton (21 DEG clusters), thylakoid (16 DEG clusters), plastid (8 DEG clusters), and cell (337 DEG clusters); and a single MF subcategory related to ligase activity (71 DEG clusters). According to the KEGG enrichment, results revealed that statistically significant differences (*P*adjust ≤ 0.05) between *P. zonale* GL and WL included 61 gene clusters assigned to ABC transport (24 DEG clusters) and the “two-component system” (37 DEG clusters) (Supplementary Table S3). 

**Figure 2:**
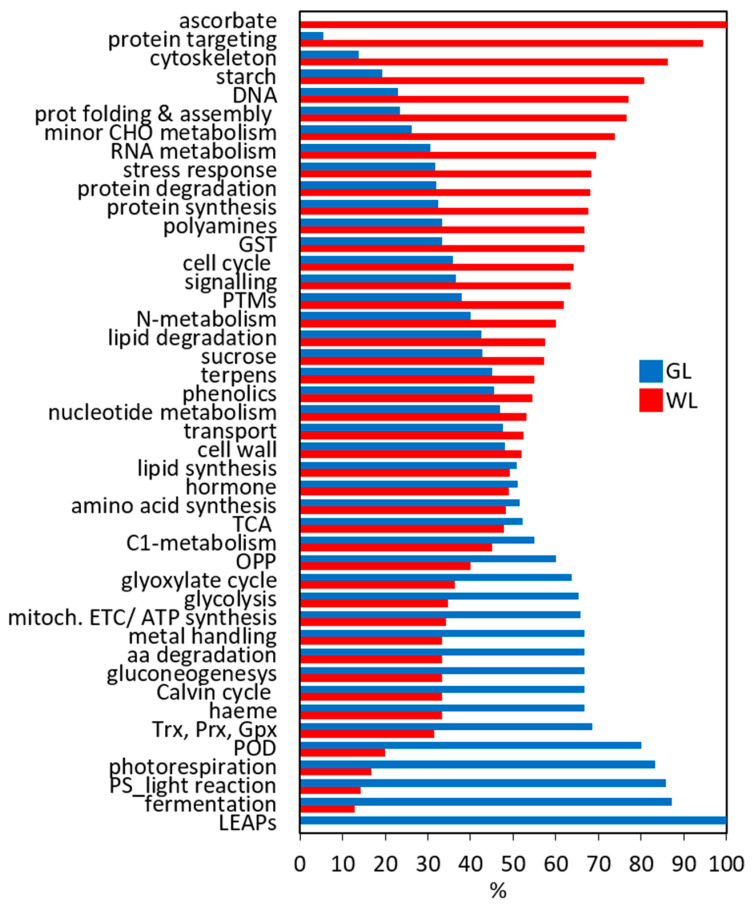
Functional classes of upregulated genes in *P. zonale* GL (blue bars) and WL (red bars). For a detailed classification of DEGs, see Supplementary Table S5. Gpx, glutathione peroxidases; GSTs, glutathione *S*-transferases; LEAPs, late embryogenesis abundant proteins; OPP, oxidative pentose-phosphate pathway; Prx, peroxiredoxins; PODs, class III peroxidases; PS, photosynthesis; PTMs, post-translational modifications; ox-red, oxidoreductases; TCA, tricarboxylic acid cycle; Trx, thioredoxins.

Independently, 3643 DEGs (~40% of the total DEGs) involved in the major metabolic pathways were manually classified into functional groups and compared (Figure 2). Interestingly, WL, although photosynthetically inactive, showed very high metabolic activity. The largest number of selected DEGs were associated with RNA metabolism (15%), transport (12%), DNA metabolism (10%), protein degradation (7.8%), signal transduction (7.5%), and general stress response (6%). All these DEGs were more upregulated in WL than in GL. All DEGs related to protein metabolism were more highly expressed in WL than in GL, including protein targeting (95%), protein folding and assembly (77%), protein synthesis (68%), protein degradation (68%), and post-translational modifications (PTMs; 62%), (Figure 2). As expected, transcripts of DEGs encoding proteins and enzymes involved in photosynthesis—light reactions and the Calvin–Benson cycle, glycolysis, and gluconeogenesis—were more abundant in GL than in WL. However, the expression levels of DEGs related to starch, ascorbate, and the cytoskeleton were higher in WL than in GL (Figure 2).

### 2.6. Major Metabolic Pathways in P. zonale Leaves

**Figure 3:**
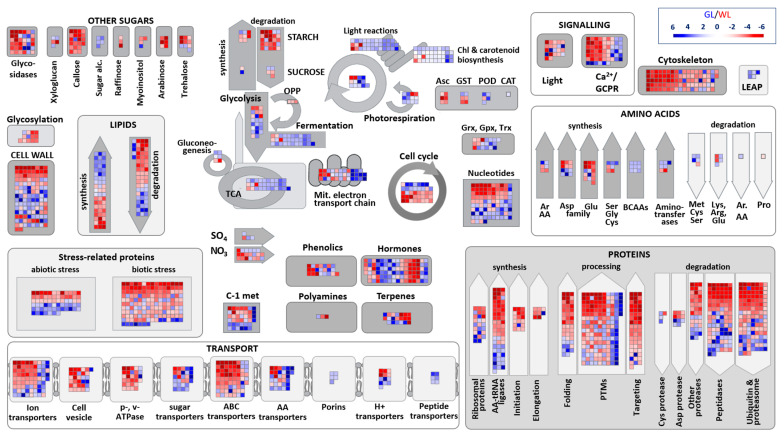
MapMan representation of DEGs in GL compared to WL of *P. zonale* manually mapped after TAIR mapping. Upregulated DEGs in GL are shown in blue (positive values), and upregulated DEGs in WL are shown in red (negative values). AAs, amino acids; ABC, ATP-binding cassette transporters; alc, alcohols; Asc, ascorbate; Ar, aromatic; BCAAs, branched-chain amino acids; C-1 met, C-1 metabolism; CAT, catalase; CHO, carbohydrates; GCPR, G-protein-coupled receptors; GSH, reduced glutathione; Grx, glutaredoxins; Gpx, glutathione peroxidases; GSTs, glutathione *S*-transferases; LEAP, late embryogenesis abundant protein; Mit, mitochondrial; OPP, oxidative pentose-phosphate pathway; POD, class III peroxidases; Prx, peroxiredoxins; PTMs, post-translational modifications; ox-red, oxidoreductases; RBPs, ribosome-binding proteins; TCA, tricarboxylic acid cycle; Trx, thioredoxins. Due to a large number of associated DEGs, DNA and RNA metabolism are not presented in this scheme.

To better understand the functional significance of DEGs, we focused on those that play a role in well-characterised biochemical pathways of photosynthetic cells using MapMan, a bioinformatics tool commonly used to visualise microarray data [33]. Figure 3 shows that DEGs are distributed across all metabolic pathways between WL and GL, but that some pathways have more DEGs than others. The most important metabolic pathways that illustrate the difference between GL and WL are explained below.

#### 2.6.1. Photosynthesis

The obvious phenotypic difference between WL and GL is reflected by the absence of chlorophyll in WL. Consequently, the majority of genes for enzymes involved in key steps of chlorophyll biosynthesis, such as delta-aminolevulinic acid dehydratase (the first step of 5-aminolevunilate to tetrapyrrole biosynthesis, Cluster-20096.45177), uroporphyrinogen decarboxylase (involved in the synthesis of coproporphyrinogen III, Cluster-20096.53756 and Cluster-20096.53736), coproporphyrinogen-III oxidase 1 (Cluster-20096.23405), and protochlorophyllide reductase (involved in divinyl chlorophyllide *a* synthesis; Cluster-20096.60419; Cluster-20096.61462; Cluster-20096.61463), and chlorophyllide *a* oxygenase, which is involved in chlorophyll *b* synthesis, were significantly repressed (up to five times; Supplementary Table S5) in WL. Among the DEGs mediating carotenoid biosynthesis, geranyl–geranyl pyrophosphate synthase (Cluster-12654.3 2.5), geranyl–geranyl transferase (Cluster-20096.36185 1.8), phytoene synthase (Cluster-20096.74171), 15-*cis*-phytoene desaturase (Cluster-20096.63979), lycopene cyclases (Cluster-20096.28909; Cluster-20096.28911), and β-carotene hydroxylases were up to three times upregulated in GL (Supplementary Table S5). Interestingly, different DEGs encoding enzymatic components of the zeaxanthin–violaxanthin cycle, zeaxanthin epoxidase (one of three), and a violaxanthin de-epoxidase (one of two) were more highly expressed in WL than in GL.

Consistent with the data in Figure 2 and Figure 3, one of the most striking alterations in the transcriptome of WL is the repression of genes for proteins that function in photosynthesis. Eleven out of fourteen DEGs encoding components of the photosystems I and II (PSI and PSII) and 19 of 21 DEGs for light-harvesting complexes (LHC) were repressed in WL in comparison to GL (Figure 3, Supplementary Table S5). Genes encoding key enzymes of the Calvin–Benson cycle and photorespiration including phosphoglycerate kinase (Cluster-20096.40227), fructose-1.6-biphosphatase (Cluster-20096.47947), fructose-bisphosphate aldolase 1 (Cluster-20096.58807), phosphoglycolate phosphatase (Cluster-20096.78633, Cluster-20096.75115), and carbonic anhydrases (Cluster-30459.0, Cluster-20096.57492) were repressed in WL. However, two out of four DEGs encoding Rubisco subunits were more expressed in WL than in GL.

#### 2.6.2. Carbohydrate Metabolism

Interestingly, our transcriptomic data showed that most DEGs for key starch biosynthesis enzymes (six of eight) were upregulated in WL (Figure 3, Supplementary Table S5). These include the only two DEGs encoding starch synthase and the only two DEGs encoding 1,4-alpha-glucan branching enzymes. Enzymes involved in starch degradation, such as α-amylase, were upregulated in GL, whereas all three β-amylase and all seven α-glucan water dikinase-related DEGs were upregulated in WL. All four DEGs encoding α,α-trehalose-phosphate synthase, which catalyses the crucial, penultimate step in the biosynthesis of trehalose, were significantly induced (2.1–9.5 times) in WL. Three transcripts corresponding to trehalosephosphate phosphatase, which dephosphorylates trehalose-6-phosphate to trehalose and inorganic phosphate, were 2–4 times induced in WL compared to GL (Supplementary Table S5).

DEGs encoding proteins involved in sucrose degradation, such as sucrose synthases (Cluster-14141.0; Cluster-20096.79556; Cluster-20096.61087), and neutral mitochondrial invertase (Cluster-20096.61972) were upregulated in WL. Of the DEGs encoding two fructokinases, one was upregulated in GL and the other in WL. No significant DEGs associated with cell wall invertases were observed.

Transcripts encoding sorbitol and mannitol dehydrogenases, involved in the degradation of sorbitol and mannitol, were downregulated in WL compared with GL. All three DEGs encoding galactinol–sucrose galactosyltransferase involved in the synthesis of raffinose were upregulated (2.3–6 times) in WL (Supplementary Table S5). Nine of twenty-five DEGs for sugar transporters, including two for sucrose transport and four SWEETIE proteins, were upregulated in WL.

#### 2.6.3. Energy Production

*Glycolysis*. Genes encoding the first- and the second-stage glycolysis enzymes, such as phosphofructokinase and phosphoglucomutase, were differentially expressed in GL and WL. All three DEGs encoding phosphoglucomutase were increased in WL, as were two of the four DEGs encoding phosphofructokinase (Figure 3, Supplementary Table S5). Two of three DEGs for pyruvate kinases were upregulated in photosynthetically active tissue. In addition, one of the three DEGs encoding phosphoenolpyruvate carboxylase (PEPC), which is involved in the conversion of PEP to oxaloacetate (OAA), was upregulated in WL compared to GL.

*Fermentation*. Even 13 out of 16 DEGs encoding alcohol dehydrogenase (ADH) and all seven DEGs encoding aldehyde dehydrogenase (ALDH) were significantly induced in GL (Supplementary Table S5).

*Oxidative and non-oxidative pentose*–*phosphate pathway*. Three of the four glucose-6-phosphate-1-dehydrogenase transcripts were upregulated in GL compared to WL. However, one transaldolase (Cluster-20096.59931) was upregulated 3.5-fold in WL (Supplementary Table S5).

*Tricarboxylic acid cycle (TCA).* Almost half of the DEGs encoding TCA cycle enzymes (52%) were induced in GL. The transcripts of three of the four citrate synthases were more highly expressed in WL (Figure 3, Supplementary Table S5). However, the level of citric acid was more than twice as high in GL (Table 1). The induction of succinyl-CoA synthetase in WL and the upregulation of all three fumarases correlated negatively with the almost nine-fold increased succinate content in GL. Both carbonate anhydrases (Cluster-30459.0; Cluster-20096.57492) were upregulated in GL.

*Mitochondrial electron transport chain (ETC)*. Most DEGs encoding proteins associated with complexes I-V were upregulated in GL (19 of 32; Supplementary Table S5). Two genes encoding uncoupling protein units (Cluster-20096.49853 and Cluster-20096.59730) were down-regulated in WL. All four DEGs encoding ATP/ADP and other mitochondrial metabolite transporters were upregulated in WL. Interestingly, all three DEGs for the alternative NADH–ubiquinone oxidoreductase, which catalyses the oxidation of mitochondrial NADH without the translocation of protons across the inner mitochondrial membrane, were induced in WL.

*Gluconeogenesis/glyoxylate cycle*. Genes mediating steps of gluconeogenesis, such as a fructose-1,6-bisphosphatase, PEP carboxykinase and enolase, were upregulated in GL compared to WL (Supplementary Table S5). As for the glyoxylate cycle, almost half of the DEGs were upregulated in WL. For example, one of two transcripts for Ala:glyoxylate aminotransferase and one for Ser hydroxyl methyltransferase were more highly expressed in WL.

In summary, relatively few genes involved in energy production, with the exception of the TCA cycle, showed twofold or greater upregulation in WL compared to GL (Figure 3).

#### 2.6.4. Lipid and Fatty Acid Metabolism

An almost equal number of DEGs involved in lipid and fatty acid biosynthesis were upregulated in WL and GL (Figure 3, Supplementary Table S5). For example, four of eight DEGs encoding acetyl-CoA carboxylase, catalysing the irreversible carboxylation of acetyl-CoA to produce malonyl-CoA which plays a key role in chain elongation in fatty acid and polyketide biosynthesis, were upregulated in WL (Supplementary Table S5). All three DEGs for malonyl-CoA decarboxylases were induced in WL, while all three 3-ketoacyl-CoA synthases were downregulated in WL. Interestingly, DEG encoding chloroplastic digalactosyldiacylglycerol synthase 1 (Cluster-20096.23718), which is involved in the synthesis of diacylglycerol galactolipids specific for thylakoid membranes, was upregulated 5.2-fold in WL. Similarly, three DEGs encoding phytyl ester synthases were 5–6 fold more highly expressed in WL than in GL. This protein has phytyl ester synthesis and diacylglycerol acyltransferase activities, which are involved in the deposition of free phytol and free fatty acids in the form of phytyl esters in chloroplasts.

The number of Induced DEGs involved in lipid degradation, such as lipases and phospholipases, and genes related to the β-oxidation of fatty acids (long-to very long-chain acyl-CoA synthetases, short-chain acyl-CoA dehydrogenases, and fatty acyl-CoA reductases), was almost equal in both leaf tissues (Figure 3, Supplementary Table S5). Despite these complex alterations in lipid metabolism, it is clear that it is impaired in WL, with a higher abundance of transcripts related to phosphoinositide and sphingolipid metabolism, while fatty acid metabolism was variably regulated in these two tissues.

#### 2.6.5. Nitrogen and Amino Acids Metabolism

Five out of six nitrate transporters (NRT1), which belong to the PTR family and are involved in the transport of dipeptides but not nitrate, were upregulated in WL compared to GL (Supplementary Table S5). On the other hand, high-affinity nitrate transporters were induced in GL. Upon the uptake, nitrate is reduced to nitrite and then to ammonium by nitrite reductase. Both DEGs encoding nitrite reductase were upregulated 2–3-fold in GL compared to WL. Both transcripts for Gln synthetase, which is responsible for the first step of nitrogen assimilation, were repressed in WL (Supplementary Table S5). Finally, all three DEGs encoding chloroplastic ferredoxin-dependent Glu synthase were upregulated in WL.

**Figure 4:**
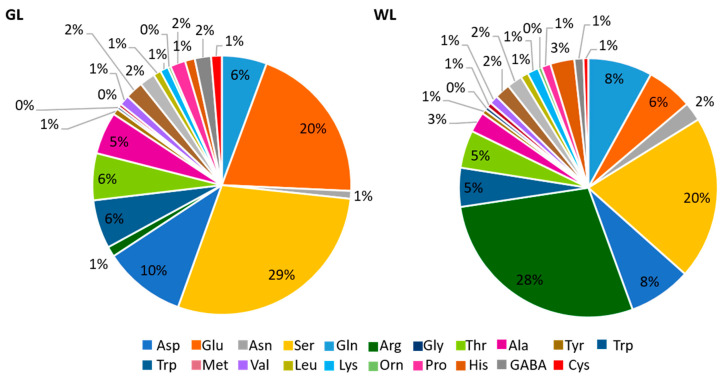
Distribution of free amino acids (AAs) in GL and WL of *P. zonale* plants. Proportions of different amino acids measured in GL (**left**) and WL (**right**) expressed in nmol g^–1^ FW are indicated.

Almost all free amino acids (AAs) were significantly more accumulated in WL than in GL (Table 1). The most abundant amino acids in GL were Ser, Glu, and Gln, while in WL, Arg, Ser, and Asp were the most abundant. The Arg content was 62 times higher in WL than in GL. The Glu AA family group contributed 37% and 44% to the total AA pool in GL and WL, respectively. Serine (29%) and Glu (20%) had the highest AA proportion in GL, in contrast to Arg (28%, compared to only 1% in GL) and Ser (20%) in WL (Figure 4). The proportion of the other AA did not differ significantly between these two tissues.

*Glu-AA family.* Genes involved in Pro biosynthesis (including four encoding delta-1-pyrroline-5-carboxylate synthase) and Glu biosynthesis were induced in WL, while those involved in Gln biosynthesis were repressed in WL compared to GL (Supplementary Table S5). DEG encoding the synthase of carbamoyl-phosphate, required for Arg synthesis from ornithine, was even 9.2-fold more expressed in GL than in WL. Similarly, DEGs related to Arg degradation were also upregulated in GL.

*Branched-chain AA family*. All nine DEGs encoding enzymes related to Val, Ile, and Leu biosynthesis were downregulated in WL compared to GL (Supplementary Table S5).

*Asp and Ser AA family.* Transcription of a large number of WL-responsive genes mediating steps in the biosynthesis of Ser- and Asp-derived AAs was differentially expressed in these two tissues (Supplementary Table S5). However, DEGs encoding enzymes involved in Met, Lys, and Asp degradation were downregulated in WL.

*Aromatic AA family.* Some of the DEGs involved in the synthesis of Phe and Tyr were repressed and some were upregulated in WL compared to GL (Supplementary Table S5). As for Trp, a DEG-encoding a subunit of Trp synthase was 2.2-fold induced in GL.

#### 2.6.6. Proteostasis

Protein content depends on its turnover and the balance between synthesis and degradation in lysosomes and proteasomes (Figure 3). Our transcriptomic data undoubtedly show increased protein translation in WL than in GL, based on upregulated DEGs encoding enzymes related to protein synthesis (62 of 87), ribosome-binding proteins (14 of 26), protein folding (36 of 47) and PTMs (114 of 184); that includes phosphorylation, dephosphorylation, and glycosylation (Figure 2, Supplementary Table S5). DEGs encoding Asp- and Gln-tRNA ligases were downregulated, while those encoding Ala-, Arg-, Asn-, Met-, Thr-, and Val-tRNA ligases were upregulated in WL. Moreover, DEGs encoding different isoforms of Pro-, His-, Cys-, Lys-, Leu-, and Gly-tRNA ligases were differentially expressed in GL and WL.

At the same time, DEGs associated with protein degradation by proteases, peptidases, and proteasome (194 out of 285) were more highly expressed in WL than in GL (Figure 2). All DEGs encoding nuclear pore complex proteins, importins, and other transporters for proteins in tonoplasts and plastids were upregulated in WL (Figure 3, Supplementary Table S5).

#### 2.6.7. RNA Metabolism

DEGs encoding proteins involved in RNA metabolism were strongly expressed in WL compared to GL. More specifically, 71% of DEGs associated with mRNA synthesis, including transcription factors (TFs), 73% of DEGs related to mRNA processing and maturation, and 68% involved in RNA regulation were induced in WL (Supplementary Table S5). Regarding TFs, GL and WL displayed different expression levels of DEGs encoding different isoforms of nuclear transcription factor Y subunits, protein plastid transcriptionally active proteins, transcription initiation factors, NAC, WRKY, WD40-like, MYB, SRM, LHW, ILR, bHLH-like, and ethylene-responsive TFs. Moreover, all TFs containing Zn-finger motifs were upregulated in WL.

#### 2.6.8. DNA Metabolism

As many as 97 out of 109 DEGs related to DNA replication and chromatin organisation were induced in WL compared to GL (Supplementary Table S5). In addition, 17 of 22 DEGs involved in DNA repair, 46 of 53 involved in histone modifications, and 20 of 24 DEGs encoding DNases were upregulated in WL compared to GL. Interestingly, out of 42 DEGs encoding reverse transcriptase, 31 were upregulated in WL, while all 16 DEGs encoding transposases were repressed in WL.

#### 2.6.9. Antioxidant Metabolism

Strong alterations of genes and proteins involved in oxidative stress responses were observed in photosynthetically inactive *P. zonale* leaves (Figure 2 and Figure 3; Supplementary Table S5). In our previous work, we characterised the major components of the antioxidant network in GL and WL under optimal conditions [20].

*General stress response and plant defence*. A significant proportion (6.5%) of the total DEGs were related to plant defence and stress response (Figure 3, Supplementary Table S5). Seventeen out of 24 heat shock proteins (HSPs) were upregulated in WL, with log2fold change ranging from 2.1 to 8.7. In addition, DEGs related to osmotic stress response, namely all seven proteins with tetratricopeptide motifs and all four HVA22-like proteins, were more upregulated in WL, by 2.2–6.1 and 2.3–5.5 fold, respectively. On the other hand, three of four DEGs encoding universal stress proteins and two DEGs encoding dehydration responsive proteins were upregulated 3.1–3.7 times in GL.

The most remarkable difference in DEGs between WL and GL relates to biotic stress. Even 87 of 95 DEGs encoding disease resistance proteins were upregulated in WL (2–8.2 times), as were all five DEGs for TMV resistance proteins (3.1–7.6 times). In addition, DEGs for four pathogenesis-related proteins (including two thaumatin-like proteins) and four Mildew Locus O (MLO)-like proteins were all more highly expressed in WL than in GL. On the other hand, four DEGs encoding endochitinases were upregulated in GL (Supplementary Table S5).

*Peroxidases*. Only five DEGs encoding class III peroxidase (POD) isoforms were detected (65 with no statistical relevance), four were upregulated in GL (2.6–4.2 times) and only one in WL (three times; Figure 3, Supplementary Table S5).

**Figure 5:**
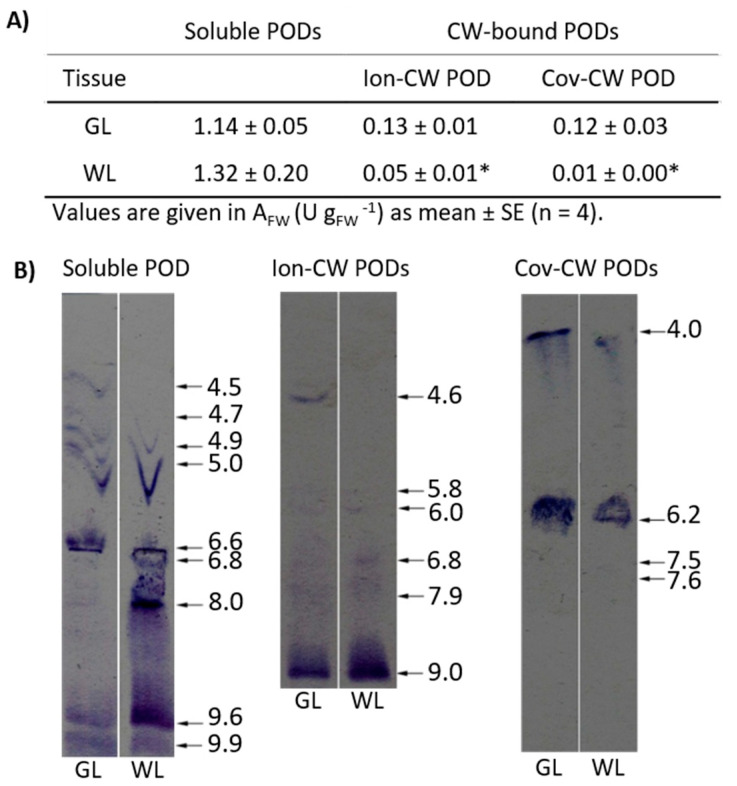
The activities and profiles of soluble, ionic, and covalent cell wall-bound peroxidases (Ion-CW POD and Cov-CW POD) in GL and WL leaf sectors of *P. zonale* plants grown under optimal conditions. (**A**) Specific POD activities (A_FW_, U g^–1^_FW_) represent means ± SE (n = 4). Significant differences between WL and GL, according to the *t*-test are indicated (* *p* < 0.05). (**B**) POD isoforms separated by IEF (pH gradient 3–10) and stained with 4-chloro-α-naphthol. The arrows indicate POD isoforms. In total, 40 µg of proteins were applied to each lane.

To better understand the physiological role of these numerous POD isoforms in these two tissues, we analysed the constitutive differences in the activities of soluble (vacuolar and apoplastic) and cell wall-related (ionically and covalently bound) PODs. As for soluble PODs, no significant differences were observed between GL and WL using guaiacol as an electron donor (Figure 5A).

To gain more detailed insight into POD isoenzyme profiles, we separated them based on their pI values. Staining gels containing soluble PODs with 4-chloro-α-naphthol over a broad pH gradient (3–10) revealed four different acidic isoforms (pI = 4.5, 4.7, 4.8, and 5.0; Figure 5B), two neutral ones (pI = 6.6 and 6.8) and three basic ones (pI = 8.0, 9.65, and 9.95). Among them, two strongly acidic isoforms were not present in WL. Instead, a strong band corresponding to an isoform with pH 8.0 was detected in WL. Of the ionically bound-cell wall PODs (Ion-CW PODs), one isoform (pI = 4.6) was present only in GL, which correlated well with the measured enzyme activity (Figure 5A). Among covalently bound-cell wall PODs (Cov-CW PODs), an isoform with pI 4.0 from photosynthetic cells showed higher reactivity with guaiacol compared to the non-photosynthetic cells, which was in agreement with the spectrophotometrically determined activity (Figure 5A).

*Ascorbate (Asc) and glutathione (GSH)*. DEG encoding L-galactono-1,4-lactone dehydrogenase (Cluster-20096.71334), which catalyses the final and committed step of Asc synthesis, was 3.4-fold repressed in GL. In addition, chloroplastic or mitochondrial ascorbate-peroxidase (APX; Cluster-20096.60992) and monodehydroascorbate reductase (MDAR; Cluster-20096.31781) were upregulated three- and seven-fold, respectively, in WL (Figure 3, Supplementary Table S5). With respect to other genes encoding the enzymatic components of the Asc-GSH cycle encoding the additional 39 APXs, seven MDARs, one dehydroascorbate reductase (DHAR), and 11 glutathione reductases (GR) were detected in *P. zonale* leaves, but these were not statistically different between GL and WL. Of the 59 genes encoding glutathione *S*-transferase (GST), four were upregulated (3–4.4 times) and two were repressed (2.5–3 times; Supplementary Table S5) in WL. Two DEGs encoding glutathione peroxidases were upregulated 2–2.5 times in GL. Four of nine DEGs for thioredoxins (Trx), and two of eight glutaredoxins (Grx) were repressed in WL compared to GL (Figure 3).

*Superoxide dismutase (SOD) and germin-like proteins*. Thirteen genes encoding FeSOD and 16 genes encoding Cu/ZnSOD were detected with a tendency towards higher expression in WL, but without statistical relevance. On the other hand, only one DEG, encoding a germin-like protein, was expressed 1.7 times more in GL than in WL

*Late Embryogenesis Abundant Proteins (LEAPs).* All five DEGs encoding LEAPs (Cluster-20096.58336; Cluster-20096.60896; Cluster-12484.0; Cluster-19485.0; Cluster-19485.1) were more highly expressed in GL than in WL (Figure 3, Supplementary Table S5). According to Pfam classification, four of them were assigned as dehydrins (Cluster-20096.58336), LEA5 (Cluster-19485.1; Cluster-19485.0), and seed maturation protein (Cluster-12484.0).

#### 2.6.10. Polyphenols

The obtained profile of phenolic compounds was generally the same in GL and WL but differed quantitatively (Table 1). Phenolics composition was similar to our previous study [1], and the profile of *P. zonale* cv. “Ben Franklin“ [34]. A comprehensive analysis of methanol extracts obtained from 58 different *Pelargonium* species, plain morphs, revealed that quercetin (Q)-based flavonols were found in all species analysed, while derivatives of kaempferol (K), protocatechuic acid (PrcA), and gallic acid (GA) were detected in at least half of the species investigated [35].

Hydroxybenzoic acids (HBAs) were the most abundant phenolics in both leaf tissues, with the highest content of *p*-hydroxybenzoic acid, GA, PrcA, and syringic acid (SyA; Table 1). Regarding the hydroxycinnamic acids (HCAs), *p*-coumaric acid (*p*-CA), and caffeic acid (CA) derivatives were detected, with the former being more abundant in WL. In addition, epicatechin and the three most abundant Q-glycosides, as well as the most abundant K-glucoside, were more accumulated in WL than in GL (Table 1).

Genes associated with polyphenol metabolism were differentially regulated; some of them were induced while others were repressed in WL (Figure 3, Supplementary Table S5). DEGs encoding enzymes involved in the shikimate and phenylpropanoid pathways, such as isochorismate synthase (Cluster-20096.13317), quinate/shikimate dehydrogenase (Cluster-20096.85200), dihydroflavonol reductase (Cluster-20096.74952), flavonol synthases (Cluster-20096.24710, Cluster-20096.77590), flavonoid 3-*O*-galactosyl transferase (Cluster-26710.0), coumaroyl-CoA:anthocyanidin-3-*O*-glucoside-6’’-*O*-coumaroyltransferase (Cluster-14162.0) were 2.3–4.7 times repressed in WL. However, DEGs for chorismate mutase (Cluster-20096.77738), shikimate dehydrogenase (Cluster-12102.0), feruloyl esterase (Cluster-20096.76549), cinnamoyl-CoA reductases (Cluster-20096.27414, Cluster-20096.71703), flavonol synthase/flavanone-3-hydroxylase (Cluster-20096.89954) were more expressed in WL than in GL (Figure 3, Supplementary Table S5).

#### 2.6.11. Cell Wall

Of the DEGs detected, nearly 60 were associated with different cell wall sugar hydrolases (Figure 3, Supplementary Table S5). Nine out of fourteen β-glucosidases were repressed (2.1–5.9 fold), while six out of eight α-glucosidases were upregulated in WL compared to GL. All three DEGs encoding fucosidases and two out of three DEGs for polygalacturonases were more highly expressed in WL. On the other hand, three out of four DEGs encoding mannosidases were upregulated in GL (2.5–5.9 times). Five out of six xyloglucan endotransglucosylase/hydrolases were up to 4.6-fold upregulated in GL. Half of the DEGs encoding cellulose synthases were upregulated in WL, while the other half were repressed. In addition, nine out of seventeen DEGs encoding pectin methylesterases (PMEs) were more highly expressed in WL (2.6–7.4 times) while four out of six PME inhibitors were more highly expressed in WL (3.3–6.8 times). A pectate lyase (PL, Cluster-20096.21415) and expansin transcripts were more highly expressed in GL than in WL (Figure 3, Supplementary Table S5). Both DEGs encoding hydroxyproline-rich glycoproteins were significantly downregulated in WL. Interestingly, 21 of 22 DEGs encoding callose synthase, involved in the synthesis of callose (β-1,3-glucan with some β-1,6-branches), were more highly expressed in WL than in GL (3.1–6.9 times), as well as six of seven β-1,3-glucanases involved in callose degradation. Two DEGs encoding laccases that catalyse the polymerisation and depolymerisation of lignin were detected (Cluster-23517.0 and Cluster-28514.0)—one strongly suppressed in WL, and one upregulated in WL.

#### 2.6.12. Cytoskeleton

DEGs encoding proteins related to the cytoskeleton, such as γ-tubulin complexes, myosins, kinesins, dynamins, and microtubule-associated proteins, were all upregulated in WL compared to GL (Figure 3, Supplementary Table S5). Overall, only 12 of 83 DEGs encoding cytoskeletal proteins were repressed in WL (including three Augmin-like proteins and six actin-related proteins).

#### 2.6.13. Metal Homeostasis

The analysis using inductively coupled plasma-optical emission spectrometry showed that transition metals—iron (Fe), copper (Cu), zinc (Zn), and manganese (Mn)—were more dominant in WL, while calcium (Ca) was more abundant in GL of *P. zonale* plants (Table 2). 

Almost half of the DEGs encoding potassium (K), magnesium (Mg), Zn, and sulphate transporters were upregulated in WL (Supplementary Table S5). Five out of six DEGs for heavy metal-associated isoprenylated plant proteins were repressed in WL, while two DEGs involved in the synthesis and transport of nicotianamine were upregulated in WL.

## 3. Discussion

The variegated pelargonium has served as a model system for a dozen fundamental studies on chloroplast development, chloroplastic DNA inheritance, chlorophyll biosynthesis, and the influence of sink tissue on the photosynthetic machinery and associated redox regulation and ROS turn-over [2,3,4,5,6,20,21,25]. Moreover, it is an excellent model system to study sink-source interactions in terms of carbon and nitrogen reallocation [1,26,27]. In this study, we used naturally variegated *P. zonale* cv. Frank Headley to gain deeper insights into the two metabolically contrasting leaf tissues.

### 3.1. De Novo Transcriptome Assembly of P. zonale WL and GL

*P. zonale* is a diploid plant with 18 chromosomes (2n) the 1C DNA value of 1.15 pg [36,37], but its genome has not yet been sequenced. Therefore, the prerequisite for further investigation of GL and WL of *P. zonale* plants was to obtain a reliable transcriptome database. Here, we provided the first *P. zonale* transcriptome database, with 139,575 annotated genes (Supplementary Table S1).

*P. zonale* belongs to clade C2 of *Pelargonium* L’Her, the second largest genus of Geraniaceae with about 280 species [38]. Clade C was the earliest branching lineage in *Pelargonium* and is distributed throughout East Africa [36]. Comparative analysis with other genomes revealed that 9% of *P. zonale* sequences were similar to the sequences of *Q. suber,* followed by *V. vinifera*, *J. regia***,** *H. brasiliensis,* and *T. cacao* (Supplementary Figure S1C). All these species belong to the clade Rosidae, while only *T. cacao* belongs to the order Malvidae as *P. zonale*. The results obtained can be explained by the large number of entries in the NCBI database, compared to the available *Pelargonium* data (https://www.ncbi.nlm.nih.gov/, accessed on 14 December 2022).

A comparable number of *P. zonale* reads was obtained from the leaves of phylogenetically and phenotypically similar *Pelargonium* × *hortorum* [36], in which, 46,475,742 trimmed reads were obtained using Illumina technology [39] (compared with 41,680,883 clean reads in GL and 45,337,775 clean reads in WL, Supplementary Table S1). Compared to the transcriptome of *P. hortorum* (of 114,762 transcripts, 42,506 were annotated, ca. 37%), a larger number of annotated transcripts (ca. 61%) was obtained for *P. zonale*.

### 3.2. Differentially Expressed Genes in P. zonale WL

Differential transcriptome analysis revealed that 62.8% of differentially expressed genes (DEGs) were upregulated in WL compared to GL. Similarly, 15% more DEGs were upregulated in the white leaf section than in the green leaf section of the variegated *Epipremnum aureum* [40]. However, a higher number of downregulated genes was reported in the white leaf sector of the Arabidopsis *var2* mutant than in the green sector [41]. In addition, a 1.5- and 2-fold higher number of downregulated vs. upregulated DEGs was detected in the white leaf sectors of the variegated *F. microcarpa* and *Hydrangea macrophylla*, respectively [17,42].

The majority of upregulated DEGs in *P. zonale* WL were related to genetic information processing (DNA replication, DNA repair, RNA transcription, homologous recombination, chromatin remodelling, histone modifications, non-homologous end-joining), protein folding, and amino acid synthesis, similar to white leaf sectors of *H. macrophylla* [42]. On the other hand, DEGs related to chlorophyll and carotenoid biosynthesis, photosynthesis, including light reactions (components of both photosystems, light-harvesting complexes, and electron carriers) were strongly repressed in *P. zonale* WL, similar to what was observed in albino tissue of other variegated species [9,16,40,42,43,44]. This is consistent with the absence of fully differentiated chloroplasts with well-organised thylakoids and starch granules in *P. zonale* WL. At the same time, the content of chlorophyll and carotenoids was significantly reduced in *P. zonale* WL tissue [45].

Therefore, the significantly increased content of Cu, Mn, and Fe in WL should not be associated with photosynthesis and the related electron transport chain. Instead, these metals could contribute as cofactors or activators of various enzymes. For example, Mn is required for the activity of RNA polymerases [46], which were upregulated in WL (39 of 56 DEGs, Supplementary Table S5). The higher Zn content in WL can be correlated with the increased number of DEGs encoding DNA-binding Zn-finger motifs (all 39 DEGs identified were induced in WL), proteins associated with DNA and RNA synthesis, reverse transcriptase, and four of six DEGs encoding Zn-transporters in WL. The higher calcium content found in GL is more likely related to the pectins of the cell wall (a higher content of galactose was found in GL, Table 1), as most DEGs encoding proteins involved in Ca^2+^-dependent signal transduction were upregulated in WL (Supplementary Table S5).

### 3.3. WL Acts as a Carbon Sink Leaf Tissue

In addition to photosynthesis and photosynthetic pigments, we have shown here that genes related to the Calvin–Benson cycle, fermentation, and glycolysis are significantly repressed in WL (Supplementary Table S5). Furthermore, our results show very little or no de novo glucose production, similar to what was observed in the white leaf sectors of Arabidopsis mutant *immutans* [16].

The electron transport chain (ETC) in mitochondria is an additional mechanism for energy production in plants, and its components were largely upregulated in GL (Figure 3). At the same time, the respiration rate was significantly lower in the non-photosynthetic leaf tissue of the variegated *P. zonale* compared to the photosynthetic one [25]. However, several proteins associated with mitochondrial ETC were upregulated in WL (Supplementary Table S5). First, Pro dehydrogenase/oxidase was induced 1.8-fold in WL. This enzyme catalyses the first step of Pro degradation while transferring electrons to the mitochondrial ETC [47]. Second, all three alternative NAD(P)H-ubiquinone oxidoreductases (AOXs) were upregulated in WL. AOXs transport electrons bypassing the complexes III and IV, without pumping protons, and therefore do not contribute to respiratory ATP production [47]. Under conditions that accelerate photorespiration, AOX accepts electrons from NADH derived from Gly oxidation allowing higher respiration rates independent of cellular adenylate status. Induced AOXs act as an alternative electron sink (a “safety valve”) to regulate the ratio of reduced and oxidised plastoquinone and prevent ROS generation under high light exposure. Increased AOX activity was found in *Nicotiana sylvestris* CMSII mutants lacking complex I under photorespiratory conditions [48]. Both genes encoding AOX in Arabidopsis were upregulated in the white leaf sectors of variegated *immutants* mutants [16]

Finally, three NADPH:adrenodoxin oxidoreductases (ADXR), the only mitochondrial FNR-like proteins, was upregulated in WL (Supplementary Table S5). Mitochondrial small iron–sulphur proteins, adrenodoxins, act as mobile shuttles that transfer electrons. The function of ADXR in plants is not fully understood. The only two roles attributed to ADXR so far are related to biotin and homocastasterone biosynthesis. The latter is required for female megagametophyte development [49].

**Figure 6:**
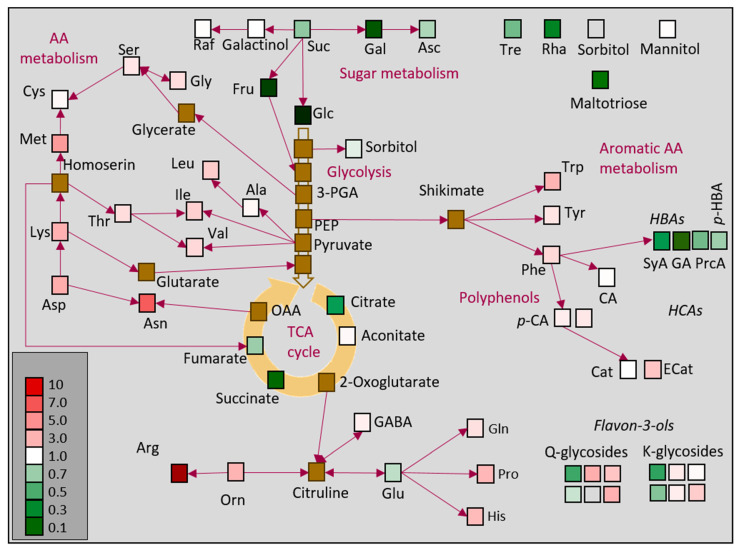
The main metabolic alterations of *P. zonale* GL and WL. The colours indicate the WL/GL for each identified metabolite. Important metabolites that were not determined are marked in brown. Asc, ascorbate; CA, caffeic acid; Cat, catechin; ECat, epicatechin; Fru, fructose; GA, gallic acid; GABA, γ-aminobutyric acid; Gal, galactose; Glc, glucose; HCA, hydroxycinnamic acids; K, kaempferol; OAA, oxaloacetate; *p*-HBA, *p*-hydroxybenzoic acid; *p*-CA, *p*-coumaric acid; 3-PGA, 3-phosphoglyceric acid; PEP, phosphoenol–pyruvate; PrcA, protocatechuic acid; Q, quercetin; Raf, raffinose; Rha, rhamnose; Suc, sucrose; SyA, syringic acid; TCA, tricarboxylic acid cycle; Tre, trehalose.

In contrast to the changes observed in the white leaf sectors of the variegated Arabidopsis *immutans* [16], several induced DEGs associated with glycolysis additionally emphasise the role of WL as metabolic sink, similar to that found in Arabidopsis tumours [50]. The concentrations of intermediates of the tricarboxylic acid cycle (TCA), such as citrate, succinate, and fumarate, were lower in WL than in GL (Table 1; Figure 6). However, the upregulation of DEGs encoding the initial enzymes of the TCA cycle contrasts with the results obtained for *immutans* [16]. The TCA cycle can be fed from different sources, such as the glyoxylate cycle and Glu metabolism, which were not repressed in WL. In addition, branched-chain amino acids (BCAAs), Pro, and Lys (all of which are more accumulated in WL than in GL, Figure 6) are considered alternative sources of respiratory substrates under stress conditions, as their oxidation directly feeds electrons into the mitochondrial ETC via the TCA cycle [51,52]. In addition, the upregulated lipases and the peroxisomal long-chain acyl-CoA synthetase might be important for providing acetate units via the glyoxylate cycle, which can be directed into the TCA cycle in the white leaf sectors.

Our previous study [1] showed higher contents of sucrose, glucose, fructose, trehalose, maltotriose, and rhamnose in GL compared to WL (Figure 6). Furthermore, efficient sucrose transport from GL to WL has been demonstrated by increased glucose and fructose content with no change in sucrose content. This fits very well with the upregulated sucrose transporters, SWEET sugar transporters, sucrose synthase, and alkaline/neutral invertase transcripts (Supplementary Table S5). Invertases have been shown to regulate carbon reallocation between sink and source tissues. In addition, three SWEETIE proteins that play critical roles in carbon allocation and utilisation were upregulated in WL. Arabidopsis with repressed SWEETIE protein accumulated significantly more sucrose, glucose, fructose, trehalose, and starch compared to wild-type plants [53].

Although the total phenolic content in WL was slightly lower than in GL, the content of flavonols and flavanols in WL was almost twice as high as in GL (Table 1). The synthesis of flavonol glycosides is more expensive than that of hydroxybenzoic acids (HBAs) in terms of reduced carbon, reducing equivalents, and ATP costs [54]. The carbon allocation from primary to more expensive secondary metabolites can account for 30% of the carbon flux and prevents photosynthesis downregulation and photoinhibition [55]. Furthermore, the more than threefold higher ratio of free amino acids (AAs) to total polyphenol content suggests that WL may act as an important energy escape valve to maintain photosynthetic performance of GL under unfavourable conditions [54].

These results confirm that WL acts as a carbon sink and depends on photosynthetic and energy-generating processes in GL. In our previous study [1], we showed that efficient sugar transport from GL to WL, stimulated by high light intensity, provides a building material for the biosynthesis of *p*-CA, K, and Q mainly in the form of glycosides. Moreover, as in the white leaf sectors of Arabidopsis mutant *immutants* [16], the abnormal plastids and altered mitochondrial ETC may act as key players in retrograde signalling leading to genome-wide changes in WL tissues. On the other hand, ROS and redox signals emanating from photosynthetically active chloroplasts (e.g., the ratio of reduced plastoquinone, hydrogen peroxide and oxidised chloroplastic metabolites) can control nuclear gene expression in the context of primary and secondary metabolism [56].

### 3.4. WL Acts as an AA Storage Compartment

Nitrate uptake was differentially regulated in these two contrasting tissues. While high-affinity nitrate transporters, which have been shown to be insensitive to nitrate concentrations [57], were induced in GL, nitrate transporters NRT1, which belong to the PRP family and are involved in dipeptide transport, were upregulated in WL. Downregulation of genes involved in nitrate uptake was described in the white leaf sectors of Arabidopsis mutants *immutants* [16]. Plastidial ferredoxin and ferredoxin reductase in non-photosynthetic tissues play a crucial role in nitrogen assimilation. All three ferredoxin-dependent Glu synthases were upregulated in *P. zonale* WL, although Glu content was slightly increased in GL (Supplementary Table S5, Table 1).

In contrast to soluble sugars, all free amino acids (AAs) except Glu, were 1.3–6.9 times more abundant in WL than in GL (Table 1, Figure 6). Higher levels of free AAs have already been reported for white leaf sectors of various variegated species, such as *H. macrophylla* [42], *P. hortorum* [27], and tea [28,43]. In *P. zonale*, the Arg content in WL was particularly high, even 62-fold compared to GL. Due to the high N/C ratio, Arg (as well as Asn and Gln) serves as a nitrogen storage compound and can function as a nitrogen transport compound. This is consistent with the previously demonstrated remobilisation of nitrogen-rich compounds from WL to GL under N deficiency in variegated *P. hortorum* [26]. Furthermore, the fivefold induction of Glu dehydrogenases in WL suggests that nitrogen released by AA catabolism can be reassimilated via Glu metabolism, as observed in the white leaf sectors of Arabidopsis *immutants* mutants [16]. On the other hand, repressed transcripts encoding Asn synthetase in WL indicate that nitrogen can be stored as Asn in *P. zonale* GL [58].

The higher content of AAs in *P. zonale* WL than in GL could be the result of (i) increased biosynthesis; (ii) decreased catabolism; and (iii) increased protein degradation. In the first case, DEGs encoding synthesis of e.g., Gln, BCAAs, and Trp were repressed in WL, although their concentrations were higher in WL than in GL. One reason for this could be the import from GL by numerous AA and dipeptide transporters induced in WL. Abadie et al. [26] showed that 50% of the ^15^N atoms taken up by GL (which were increased by the presence of the white tissue) were actually exported to WL of variegated *P. hortorum*. Moreover, a discrepancy between the transcript abundance (both in chloroplasts and in the nucleus) and the corresponding enzymes and subsequently their products have been observed [59]. This can be attributed to dynamic post-transcriptional regulation, mRNA half-lives, protein turnover, and post-translational modifications (PTMs) [60,61]. The second reason for higher levels of AAs in WL may be due to their lower consumption, e.g., reduced chlorophyll biosynthesis (starting from Glu). On the other hand, as mentioned above, Pro, BCAAs, and Lys contribute to the TCA cycle, which can be considered as a compensatory mechanism for the low glucose content in WL. Third, enhanced protein degradation, either via peptidases and proteases, such as Asp, Ser, Met, and Cys-proteases, metalloproteases, three subtilisin-like proteases, or via the ubiquitin-dependent proteasome degradation pathway, was indeed upregulated in WL. This could be one reason for the higher level of free AAs in WL. However, a higher number of DEGs encoding proteins involved in RNA transcription and processing, specific AA-tRNA ligases, protein translation and folding, and PTMs (which may involve proteases such as subtilisin) were also found to be upregulated in WL than in GL (Supplementary Table S5). The significant increase in AA content has been attributed to protein degradation in Arabidopsis and tea chlorotic mutants [43,62].

Overall, we have provided additional evidence that nitrogen metabolism compensates for insufficient energy for carbon metabolism. Indeed, under conditions of limited carbohydrate supply, as in WL, plants can metabolise proteins as alternative respiratory substrates [51]. In addition, *P. zonale* WL can serve as nitrogen storage [26,43].

### 3.5. WL Has an Efficient Protective System against (a)Biotic Stress

In our previous studies [1,20,21], we demonstrated an efficient antioxidant network in *P. zonale* WL, especially with respect to H_2_O_2_ scavenging. Although a whole battery of genes encoding Fe superoxide dismutase (FeSOD), Cu/ZnSOD, the ascorbate–glutathione (Asc-GSH) cycle constituents, and class III peroxidases (PODs) was detected in *P. zonale* leaves, only several were identified as statistically significant DEGs (Supplementary Tables S2 and S5). In fact, only a few genes encoding enzymatic components of the Asc-GSH cycle were differentially expressed, including the chloroplastic ascorbate peroxidase (APX) and monodehydroascorbate reductase (MDAR), which were upregulated in WL (Supplementary Table S5). In the absence of photosynthetic machinery involved in light reactions, these enzymes are involved in the detoxification of H_2_O_2_ generated by other mechanisms. Consistent with the elevated levels of Gly, Cys, and glutathione in WL, four of the six glutathione *S*-transferases (GST) were upregulated in WL. Similarly, lower levels of ROS and higher content of glutathione were found in the white leaf tissue of variegated *E. aureum* [40]. The white leaf tissue of variegated *Pittosporum tobira* has a much more efficient ROS-scavenging machinery compared to the green leaf tissue and shows lower lipid peroxidation [43]. Consistent with our CAT-activity measurements [20], the absence of peroxisomes [1], and transcriptomic analysis of white and green leaf sectors of Arabidopsis mutant *immutans* [16], DEG encoding CAT was suppressed in WL (Supplementary Table S5).

Although the overall activity of all soluble POD isoforms was similar in WL and GL, a higher number of upregulated genes encoding POD isoforms was found in GL than in WL (Supplementary Table S5). This was consistent with the higher activity of cell wall-bound POD isoforms, both ionic and covalent, and the additional isoforms detected in GL (Figure 5). The clear tendency of POD activities between green and white leaf tissues of the two variegated Arabidopsis mutants *var2* and *immutants* was not obtained, as several POD isoforms were upregulated in WL, while some were upregulated in GL [16,41]. On the other hand, as mentioned above, significantly higher contents of *p*-CA, epicatechin, and several Q- and K-glycosides (which have been shown to act as endogenous POD co-substrates, Vidović et al. [20]) were found in WL (Figure 6). It is possible that these polyphenols play a role in direct ROS scavenging and prevention of lipid peroxidation, as suggested by Agati et al. [63]. An accumulation of *ortho*-dihydroxy B-ring substituted phenylpropanoids (CA, Q, Cat, and cyanidin, Cy) with higher antioxidant potential [64] at high light intensity has been demonstrated in *P. zonale* plants [1].

Given the absence of photosynthetic ETC and peroxisomes in WL, the subcellular localisation of the enzymatic components of the cellular H_2_O_2_ regulatory network is crucial for characterising the antioxidant system in WL. This is particularly important since antioxidants in WL can respond rapidly to accelerated superoxide and H_2_O_2_ production in GL [21]. Moreover, under accelerated Mehler reaction, NADPH oxidase-dependent H_2_O_2_ accumulation was observed exclusively in the apoplast of GL bundle sheet cells, but not in WL. Therefore, possible communication between neighbouring GL and WL cells could occur via H_2_O_2_-mediated signalling. Communication between the adjacent cells could be achieved through plasmodesmata, as well. Cell-to-cell transport via plasmodesmata is regulated by callose deposition [65]. Interestingly, *P. zonale* WL showed upregulated callose metabolism (Supplementary Table S5). The yellow leaf sectors of the variegated *tie-dyed2* (*tdy2*) maize mutants exhibit defective callose synthase Tdy2, which is important for sugar transport across the plasmodesmata to the phloem [66]. Nevertheless, the investigation of long-distance signalling between WL and GL should be a goal of future studies using *P. zonale* as a model system.

Among other stress-responsive genes, DEGs encoding proteins related to heat, abscisic acid (ABA), and osmotic stress were also upregulated in WL tissues (Supplementary Table S5). A similar situation was observed in the white leaf sectors of Arabidopsis *immutants* mutants [16] and in the barley mutant *albostrians*, where several stress- and pathogenesis-related genes were induced in the white leaf sectors compared to the wild type [67]. In addition to the aforementioned role in regulating transport across plasmodesmata, callose forms a protective barrier in the cell wall under different (a) biotic stresses [65]. Another defence-related compound, Pro, was 1.7 times more accumulated in WL than in GL of *P. zonale*. Similar to the white leaf sectors of Arabidopsis *immutants* mutants, genes involved in Pro biosynthesis were also induced in *P. zonale* WL (Supplementary Table S5). In addition to its osmoprotective function, Pro has several protective functions: stabilisation of the peptide backbone and prevention of protein aggregation, cell wall reinforcement by (hydroxyl-) proline-rich proteins and ROS-scavenging. In parallel, with the aforementioned protective strategies, DEGs encoding heat shock proteins (HSPs), chaperones, and DNA repair genes to protect important cell components from oxidative stress were upregulated in *P. zonale* WL (Supplementary Table S5), similar to what was observed in the white leaf section of variegated *P. tobira* and *E. aureum* [40,43]. On the other hand, dehydration-responsive proteins and desiccation-inducible late embryogenesis abundant (LEA) proteins were upregulated exclusively in GL.

The significant upregulation (93 out of 101) of DEGs encoding proteins involved in biotic stress response in WL was one of the major findings of this study. Proteins associated with pathogen attack were also upregulated in the white leaf sectors of Arabidopsis *immutants* mutants [16]. In the case of the variegated *H. macrophylla*, where a higher abundance of transcripts involved in plant–pathogen interactions were also observed, the hydrangea ringspot virus replicates in the white leaf tissue without invading the green leaf tissue [42].

Hess et al. [67] suggested that the upregulation of many of these defence-related genes in WL could be a response to the highly altered metabolism in WL cells, which can be considered stressed by multiple stressors. As a result, these cells might re-regulate their transcriptome and activate different protective systems to cope with stress, particularly in mutants in which the albino phenotype is related to the photooxidation of chlorophyll and the inability to repair PSII [40,41]. On the other hand, WL, as mentioned above, can generate its own, GL-independent signals, that can be interrelated with H_2_O_2_ or other (retrograde) signalling molecules and transmitted to GL.

### 3.6. Abnormal Metabolism in P. zonale WL Plastids

In addition to photosynthetic pigments, a hallmark of the white/yellowish leaf sectors of the variegated leaves is the absence of fully differentiated chloroplasts. As already mentioned, the chloroplasts of *P. zonale* WL lack developed thylakoid membranes organised into grana and stroma.

A thylakoid-bound ATP-dependent zinc metalloprotease FtsH (member of the AAA protease subfamily) plays an important role in thylakoid development. Knockdown of the FtsH2 subunit results in variegation phenotype of *yellow variegated 1 and 2* Arabidopsis (*var2*) mutant [12,13]. Contrastingly, in *P. zonale* WL, ten out of twelve DEGs encoding FtsH subunits were upregulated up to five-fold, as were all four proteins containing an ATPase family AAA domain (Supplementary Table S5).

In rice, disruption of two pentatricopeptide repeat (PPR) proteins leads to a transient albino phenotype [68]. These proteins (encoded by nuclear genes) play a central and extensive role in modulating the expression of organellar genes at the post-transcriptional level (RNA editing and splicing), plastid ribosomes biogenesis, and retrograde signalling [69]. However, in *P. zonale* WL, even 42 out of 51 PPR proteins were upregulated (Supplementary Table S5), indicating the importance of maintenance and editing of chloroplastic and mitochondrial transcripts, as well as retrograde signalling to the nucleus.

Even 11 genes encoding the FtsZ protein, a regulator of chloroplast division that was downregulated in *Anthurium andraeanum* mutant leaves [70], were detected in *P. zonale* leaves, but without statistically relevant differences between GL and WL. The cytoskeleton’s tubulin-like GTPase, FtsZ, activates the bundling of protofilaments that form the midcell FtsZ ring [71]. Concurrent with the alteration in filament and microtubule architecture, a significant number of DEGs encoding motor proteins, i.e., kinesin, myosin, and dynein, were upregulated in WL (Supplementary Table S5). They move along microtubules, intermediate and actin filaments, and are important not only for chloroplast development, movement, and division, but also for cell division, cell wall remodelling, and transport of organelles and vesicles [72].

In addition, the transcription factor (TF) Golden 2-like1 (GLK1-like; belongs to the GARP TF family) regulates chloroplast development and synchronises the expression of chlorophyll biogenesis genes, [7]. Similar to the variegated *A. andraeanum* [70], a single DEG encoding GLK1-like was repressed in *P. zonale* WL (Supplementary Table S5).

The gene responsible for the variegated phenotype in the *albostrians* barley mutant is *CCT Motif Family gene 7* (the homologue of Arabidopsis *CONSTANS* TF) [73], which is involved in chloroplast biogenesis. Two DEGs encoding the CONSTANS-like TF were repressed in *P. zonale* WL, and two were strongly induced.

Furthermore, two DEGs encoding mitochondrial transcription termination factors (mTERF) were upregulated two-fold in *P. zonale* WL, while they were significantly downregulated in the white leaf sectors of variegated *F. microcarpa* [17]. It has been suggested that mTERF is responsible for the albino phenotype in *F. microcarpa*, as it is involved in the regulation of chloroplast development [74].

Overall, there is no clear overlap between the abovementioned genes responsible for impaired chloroplast biogenesis and those repressed in *P. zonale* WL. However, plastids in *P. zonale* WL showed highly altered metabolism, compared to that of typical chloroplasts in GL. The modulation of the expression levels of these key genes encoding chloroplastic proteins may be orchestrated by different TFs that are, in general, upregulated in WL (Supplementary Table S5).

Although plastids in *P. zonale* WL did not evolve into chloroplasts, specific DEGs related to the synthesis of fatty acids and certain AAs, phenylpropanoids, and terpenoids (such as phylloquinone and ABA) were even upregulated in WL (Supplementary Table S5). Intriguingly, one of the most surprising results regarding the identified DEGs was related to starch synthesis and degradation, which were upregulated in WL (Figure 3), as starch was not detected in plastids of the mesophyll tissue of *P. zonale* white leaf sectors [1]. Plastids in the yellow leaf tissue of variegated *P. tobira* contain starch grains, as well as a high content of carotenoids [43]. In addition, enzymes important for chloroplastic metabolism, such as phytyl ester synthases and chloroplastic digalactosyldiacylglycerol synthase 1, which is required for thylakoid development and function, were strongly upregulated in *P. zonale* WL (Supplementary Table S5). Moreover, two proteins LOW PSII ACCUMULATION and two chlorophyll *a-b* binding proteins were upregulated in WL. As for the stroma, two DEGs encoding large subunits of Rubisco and two DEGs encoding the components of the zeaxanthin–violaxanthin cycle were upregulated in WL, as well.

A possible explanation for these unexpectedly upregulated genes could be the contribution of the stomatal guard cell transcriptome to our de novo assembled transcriptome of *P. zonale* WL. As a periclinal chimaera, WL of variegated *P. zonale* cv. Frank Headley exhibits a mutation in the L2 layer of the meristem [3]. During organogenesis in dicotyledonous flowering plants, the L2 layer forms most of the mesophyll tissue of the leaves, while the L1 layer gives rise to the epidermis [2]. In WL of *P. zonale* cv. Frank Headley, only the stomatal cells contain normally organised and developed chloroplasts [45]. Mentioned unexpectedly upregulated DEGs could therefore encode specific isoforms of the respective proteins present in the stomatal cells. Despite this observation, the level and number of starch-related DEGs are still above expectations, the explanation of which requires further investigation.

## 4. Materials and Methods

### 4.1. Plant Material and Growth

The model plant used in this experiment was the variegated *P. zonale*, cultivar “Frank Headley” [1]. The stock plants were purchased one year before the experiment from nurseries Silze GmbH & Co. Weener-Halte, DE, and Raritätengärtnerei Fam. Treml, Arnbruck, DE. Plants were vegetatively propagated in pots with Substrate 2 (Klasmann-Deilmann, Geeste, Germany) and grown in the growth chamber at a day/night temperature of 25/22 °C with a 16-h photoperiod (06:00–22:00, Central European Time, CET) and 230–280 µmol m^–2^ s^–1^ of photosynthetically active radiation (PAR). The plants were regularly watered.

### 4.2. RNA Extraction, cDNA Library Construction, and Illumina High-Throughput Sequencing

The de novo transcriptome of GL and WL of *P. zonale* plants grown under physiological growth conditions was assembled. For the construction of *P. zonale* transcriptome, high-quality RNA from GL and WL was extracted according to our previously optimised TRIzol-based protocol [32]. Extractions were performed from three biological replicates (G1-G3, and W1-W3, each representing a mixture of four plants, three mature leaves per plant). The quality and quantity of the total RNA was determined using the RNA Nano 6000 Assay Kit on the Agilent Bioanalyzer 2100 system. One µg of total RNA per sample was used for poly-T oligo-attached magnetic beads-based purification of mRNA, and cDNA library construction was performed using the NEBNext Ultra RNA Library Prep Kit for Illumina (NEB, USA), described in detail in Vidovic and Cukovic [32]. Clustering of index-coded samples was performed on a cBot Cluster Generation System using the PE Cluster Kit cBot-HS (Illumina) according to the manufacturer’s instructions. Paired-end sequencing was performed on the Illumina HiSeq 4000 platform (Illumina, San Diego, CA, USA).

### 4.3. Transcriptome De Novo Assembly

The raw reads from Illumina were processed into clean data using in-house scripts to remove low-quality reads, reads containing adapter sequences, poly-N sequences, and contaminants. The resulting high-quality clean reads were subjected to de novo transcriptome assembly using Trinity [75] (using min_kmer_cov = 2 and min_glue = 2, while other parameters were used by default). The redundancy from the Trinity results was removed using Corset software, which hierarchically clusters transcripts based on multiple mapping events and expression patterns [76]. The longest transcripts in each cluster (contigs filtered by Corset) were selected as the unigenes.

The raw data from this article can be found in the NCBI Short Read Archive database (https://www.ncbi.nlm.nih.gov/sra, accessed on 24 December 2022) under accession numbers SRX14189101, SRX14189100, SRX14189099, SRX14189098, SRX14189097, and SRX14189096 (three GL samples and three WL samples) and Bioproject accession no. PRJNA807121 and Sample accession no. SAMN25962180—WL, and SAMN25962179—GL).

### 4.4. Gene Functional Annotation

Functional annotation of the unique assembled transcripts was performed using the following databases: NCBI non-redundant protein sequences (Nr), NCBI non-redundant nucleotide sequences (Nt), Protein family (Pfam) database [77], Clusters of Orthologous Groups of proteins (KOG/COG), Swiss-Prot, Kyoto Encyclopedia of Genes and Genome (KEGG) Ortholog database [78], and Gene Ontology (GO). Based on the results from BLAST, the coding sequence (CDS) was either extracted directly from the unigene sequences and translated into peptide sequences using the standard codon table or predicted using ESTScan.

### 4.5. Differential Expression Analysis and Functional Enrichment

Gene expression levels were estimated using RSEM [79] for each sample by mapping clean data back to the assembled transcriptome (using Bowtie2 with mismatch = 0) and counting reads for each gene from the obtained mapping results. RSEM converts read counts into FPKM (Fragments per Kilobase of transcript sequence per Million base pairs sequenced) values, taking into account the effects of sequencing depth and gene length on the fragments counting.

To identify differentially expressed genes (DEGs) between *P. zonale* GL and WL, the expression levels of individual transcripts were compared between samples (or sample groups). Differential expression analysis between GL and WL (three biological replicates for each tissue type) was performed using the DESeq2 R package. The resulting *p* values were adjusted using the Benjamini and Hochberg’s approach for controlling the False Discovery Rate. Genes with abs (log2 FC) ≥ 2 and an adjusted *p* value < 0.05 found by DESeq2 were assigned as statistically significant differentially expressed.

Functional enrichment analysis included GO, KOG, and KEGG terms enrichment. GO enrichment analysis of differentially expressed genes was performed using the GOseq R package, in which gene length bias was corrected. Statistical enrichment of differentially expressed genes in KEGG pathways was tested using KOBAS software [80]. GO, KEGG, and KOG terms with corrected *p* < 0.05 were considered significantly enriched by DEGs.

Differentially abundant proteins (predicted by translating the CDS of functionally enriched differentially expressed genes into amino acid sequences) were mapped to metabolic pathways using MapMan 3.6.0RC1 software [33]. Functional classification of the obtained DEGs using the TAIR codes was performed manually.

### 4.6. Extraction and Analysis of Amino Acids

GL and WL material (approximately 100 mg) was homogenised with liquid nitrogen using steel beads (3 mm) and the vibratory mill TissueLyser II (Qiagen) at a frequency of 30 Hz for 100 s to a fine powder. The homogenised leaf tissue was mixed with 800 µL of 50% methanol and 800 µL of chloroform, shaken for 1 h at 4 °C in the dark, and centrifuged for 10 min at 16,000× *g* at 4 °C. The upper water–methanol phase was removed and dried in a speed-vac. The obtained pellet was resuspended and mixed with 0.5 M sodium borate buffer (pH 9.5), 0.4 M *ortho*-propionic acid (OPA), and β-mercaptoethanol (9:1:0.2, *v*:*v*:*v*) for amino acid (AA) derivatisation.

The obtained AA derivatives were identified and quantified using an HPLC instrument (LC-20AB Prominence Liquid Chromatograph, Shimadzu, Kyoto, Japan) coupled with an RF-10-AXL fluorescence detector. The excitation wavelength was set to 340 nm and the emission wavelength to 450 nm. The AA derivatives were separated on onto a 5.0 µm, 250 × 4.6 mm Luna C18(2) reversed-phase column (Phenomenex Ltd., Torrance, CA, USA) at 40 °C and flow rate of 1.1 mL min^−1^. The elution gradient was formed with eluent A: 20 mM sodium phosphate buffer pH 6.8: methanol: tetrahydrofuran (THF; 90:9:1, *v:v:v*) and eluent B (20 mM sodium phosphate buffer pH 6.8: methanol: THF, 40:59:1). The following elution gradient was used: 0–5 min A = 100%, B = 0%; 5–15 min A = 85%, B = 15%; 15–30 min A = 85%, B = 15%; 30–40 min A = 40%, B = 60%; 40–40.01 min A = 20%, B = 80%; 40.01–45 min A = 20%, B = 80%; 45–52 min A = 0% B = 100%; 52–60 min A = 100% B = 0%. The injection volume was 20 µL. Quantification was based on peak area using Shimadzu LC Solution software (Shimadzu, Kyoto, Japan).

Proline content was measured spectrophotometrically according to Bates et al. [81] with minor changes. Frozen leaf sectors were homogenised in liquid nitrogen, extracted in 3% sulphosalicylic acid (1:10, *w*:*v*), and centrifuged at 14,000× *g* for 10 min at 4 °C. The supernatant was mixed with equal volumes of acid ninhydrin and glacial acetic acid and incubated at 100 °C for 60 min. The reaction was stopped in the ice bath and toluene was added in 1:1 (*v*:*v*) ratio. The upper organic phase was used for the determination of Pro by absorbance at 520 nm.

### 4.7. Polyphenol Analysis

The frozen GL and WL material was rapidly homogenised in liquid nitrogen, extracted in methanol containing 0.1% HCl, and then acid hydrolysed to determine aglycones, as previously described in Vidović et al. [1]. Extractions were performed in duplicate and the obtained extracts and re-extracts were flushed with nitrogen.

For HPLC analysis, samples were loaded onto a 5.0 µm, 250 × 4.6 mm Luna C18(2) reversed-phase column (Phenomenex Ltd., Torrance, CA, USA) mounted in a UPLC system coupled to a photodiode array detector (Ultimate 3000, Thermo Fisher Scientific, USA). The phenolic compounds were separated at a flow rate of 1 mL min^−1^ with a mixture of solvent A (acetonitrile) and solvent B (acetic acid/acetonitrile/phosphoric acid/water: 10.0:5.0:0.1:84.9, *v*:*v*:*v*:*v*) at 25 °C, as described in Vidović et al. [1]. The following elution procedure was used to achieve separation of a wide range of phenolics: 0–5 min, 100% solution B (isocratic step); 5–25 min, 100–80% solution B (linear gradient); 25–35 min, 80–60% solution B (linear gradient); 35–40 min, 60–100% solution B (linear gradient). Phenolics were analysed using the SPD-M20A diode array Prominence (Shimadzu). Chromatograms were recorded at different wavelengths depending on the characteristic absorption maximum of the selected phenolics: 520 nm for anthocyanins, 340 nm for flavones, 320 nm for HCAs and their derivatives, and 280 nm for catechins, hydroxybenzoic acids (HBAs), and their derivatives. The individual phenolics were identified by comparing the absorption spectra with authentic standards and by spiking. Quantification was based on peak area using Chromeleon CDS 6.8 software (Thermo Fisher Scientific, Sunnyvale, CA, USA).

### 4.8. Organic Acid Analysis

The frozen GL and WL material was rapidly homogenised in liquid nitrogen and extracted in 75% methanol and centrifuged at 10,000× *g* for 10 min at 4 °C. The obtained pellet was re-extracted, and the extracts and re-extracts were pooled together. The extractions were carried out in duplicate.

Organic acids were analysed by HPLC coupled to a photodiode array detector (LC-20AB Prominence liquid chromatograph, Shimadzu, Kyoto, Japan) on a Rezex ROA organic acid column (300 × 7.8 mm, 8 μm; Phenomenex, Torrance, CA, USA) maintained at 25 °C. Isocratic elution system (95% of 5 mM H_2_SO_4_ and 5% of acetonitrile) with stepwise flow rate gradient: 0.2 mL min^−1^ in the first 45 min and 0.5 mL min^−1^ from 45 to 60 min [82]. Organic acids were detected at 210 nm and identified by comparing the absorption spectra with authentic standards and by spiking. Quantification was based on peak area using Shimadzu LC Solution software (Shimadzu, Kyoto, Japan).

### 4.9. Peroxidase Extraction and Activity Measurements

The frozen GL and WL material was homogenised in a mortar with pestle and liquid nitrogen. Soluble class III peroxidases (PODs, EC 1.11.1.7) were extracted with 100 mM sodium phosphate buffer (pH 7.0) containing 0.1% (*w*/*v*) Triton X-100 and 1 mM EDTA containing 5% (*w*/*v*) insoluble polyvinylpyrrolidine (PVP) and 5% (*w*/*v*) Protease Inhibitors Cocktail (P2714; Sigma-Aldrich, St. Louis, MO, USA).

Cell wall isolates were obtained from separated GL and WL material following the protocol described in Kukavica et al. [83]. Briefly, the leaf material was homogenised in 50 mM TRIS buffer pH 7.2, 50 mM NaCl, and 0.05% Tween-20 containing 1 mM phenylmethylsulphonyl fluoride at 4 °C. The homogenate was centrifuged at 1000× *g* for 20 min and the pellet was washed four times in 50 mM TRIS buffer, pH 7.2. To extract the ionically bound cell wall protein fraction, the pellet was suspended in 1M NaCl, incubated at 4 °C for 30 min, and then centrifuged at 1000× *g* for 15 min [83]. The supernatant was used for analysis of ionically bound POD, while the pellet was washed four times with 50 mM TRIS buffer pH 7.2 containing 50 mM NaCl and 0.05% Tween-20. The covalently bound cell wall protein fraction was released after incubating the pellet with 0.5% cellulase and 2.5% pectinase at 4 °C for 24 h and centrifuged at 1000× *g* for 15 min. The supernatant obtained was used for the analysis of the covalently bound-cell wall PODs.

The POD activity was determined spectrophotometrically using guaiacol as an electron donor, by the increase in absorbance at 470 nm (ε_470_ tetraguaiacol = 26.6 mM^–1^ cm^–1^) [20]. The POD activity was determined in triplicate at 25 °C, using a temperature-controlled spectrophotometer (UV-160; Shimadzu, Kyoto, Japan). The protein content in the samples was determined according to Bradford [84].

### 4.10. Isoelectric Focusing of POD Isoforms

Isoelectric focusing (IEF) of POD isoforms from GL and WL of *P. zonale* plants was performed with 7.5% (*w*/*v*) polyacrylamide and 3% ampholyte mixture in a pH gradient of 3 to 10 [20]. The total amount of proteins applied to each line was 40 µg. The active POD isoforms were visualised by staining with 0.01% 4-chloro-α-naphthol according to Vidovic et al. [20].

### 4.11. Metal Determination

For the determination of Mn, Ca, Zn, Fe, and Cu content in GL and WL of *P. zonale*, all samples were dried at 70 °C for 24 h and then milled. The ground samples (approximately 100 mg) were digested in 3 mL 69% HNO_3_ and 2 mL 30% H_2_O_2_ using a microwave digestion system. Metal content was determined by inductively coupled plasma–optical emission spectrometry (ICP-OES).

### 4.12. qPCR

Plant tissue was frozen in liquid nitrogen and ground using mortar and pestle. Total RNA was extracted from 300 mg of WL and GL according to the CTAB-based protocol described in Vidović and Ćuković [32]. To remove any DNA remaining prior to cDNA synthesis, total RNA samples were treated with the Ambion^®^ DNA-free™ DNase Treatment and Removal DNA Kit. The cDNA synthesis was performed according to the Thermo Fisher Scientific protocol using Random Hexamer Primer and the RevertAid™ Reverse Transcriptase.

Prior to the SYBR Green assay, total cDNAs were diluted 1:4 with nuclease-free water. Reactions were performed in a volume of 25 µL contained 300 nM of each primer and 1X SYBR Green PCR Master Mix (Thermo Scientific). Real-time PCR was performed on the Mic Real Time PCR Cycler (Bio Molecular Systems) with the following cycles: 2 min at 50 °C, 10 min at 95 °C and 40 cycles of 95 °C for 15 s, 60 °C for 1 min. Each PCR reaction was performed in duplicate and no template control was used. Amplification of PCR products was detected in real time and results were analysed using micPCR software (Bio Molecular Systems) and presented as 2^−dCt^. Primers used for gene expression analysis were designed using Primer 3 software based on de novo transcriptome sequencing. Primer pairs (Supplementary Table S4) included the genes related to antioxidant metabolism: *APX,* ascorbate peroxidase; class III peroxidase isoforms *POD17, POD42,* and monodehydroascorbate reductase, *MDAR*; and *CHS;* dihydroflavonol-4-reductase, *DFR*. The values of the relative gene expression changes were calculated by applying actin gene reference.

### 4.13. Statistics

To test for significant differences in the analysed metabolites and metals between GL and WL, the *t*-test was used and the significance threshold was set at 0.05. All analyses were performed using the statistical software IBM SPSS (version 20.0, SPSS Inc., Chicago, IL, USA).

## 5. Conclusions

This study presents the first de novo transcriptome analysis of variegated *P. zonale*. Our results revealed the following differences between WL and GL:

1. WL acts as a carbon sink and depends on photosynthetic and energy-generating processes in GL;
2. WL can serve as a nitrogen storage for GL;
3. Upregulated nitrogen and protein metabolism in WL might provide alternative respiratory substrates;
4. WL exhibited upregulated H_2_O_2_ scavenging network, protein and DNA repair and pathogen defence system;
5. Genes encoding motor proteins associated with cell division, DNA replication, modification, repair, and recombination were induced in WL.

Overall, our study provides a new genetic data resource for further research with this excellent model system and for ornamental pelargonium breeding. Furthermore, it contributes to uncovering molecular genetic mechanisms underlying foliar variegation and understanding its adaptive value, as no consistent conclusions on its ecological benefits have been proposed so far.

## Figures and Tables

**Table 1:** Content of selected metabolites in GL and WL of *P. zonale*.

Metabolite	GL	WL	Metabolite	GL	WL
*Organic acids (µmol g^–1^ FW)*			*Amino acids (nmol g^–1^ FW)*		
Oxalic acid	1039 ± 9	1911 ± 38 **	Aspartic acid	358 ± 28	1433 ± 75 **
Citric acid	510 ± 30	221 ± 3 *	Glutamic acid	1277 ± 104	1029 ± 65
Tartaric acid	439 ± 20	612 ± 4 *	Asparagine	62 ± 9	420 ± 87 *
Aconitic acid	229 ± 51	292 ± 6	Serine	1832 ± 266	3637 ± 260 *
Formic acid	72 ± 14	379 ± 16 ***	Glutamine	654 ± 87	1402 ± 158 *
Malic acid	1075 ± 239	620 ± 14	Arginine	81 ± 4	5022 ± 770 **
Fumaric acid	3.7 ± 0.1	2.6 ± 0.4	Glycine	386 ± 54	868 ± 97 *
Succinic acid	90 ± 9	11 ± 1 *	Threonine	372 ± 23	840 ± 74 *
Ascorbate	2.0 ± 0.1	1.5 ± 0.1 **	Alanine	335 ± 45	453 ± 35
*Hydroxybenzoic acids (µmol g^–1^ FW)*			Tyrosine	50 ± 7	100 ± 13 *
*p*-HBA	49.1 ± 4.3	34.9 ± 3.2 *	Tryptophan	21 ± 1	78 ± 7 *
Gallic acid	4.5 ± 0.3	1.5 ± 0.1 ***	Methionine	24 ± 3	110 ± 8 *
SyA	1.5 ± 0.2	0.6 ± 0.1 ***	Valine	81 ± 10	194 ± 10 *
PrcA	2.4 ± 0.2	1.4 ± 0.1 ***	Phenylalanine	142 ± 5	346 ± 49 *
*Hydroxycinnamic acids (µmol g^–1^ FW)*			Isoleucine	124 ± 11	338 ± 53 *
*p*-CA	1.2 ± 0.2	2.0 ± 0.3 **	Leucine	60 ± 11	172 ± 12 *
Caffeic acid	0.3 ± 0.1	0.3 ± 0.1	Lysine	65 ± 9	247 ± 32 *
*p*-CA derivative	1.2 ± 0.2	2.3 ± 0.3 **	Ornithine	22 ± 2	82 ± 9 *
*Flavan-3-ols (µmol g^–1^ FW)*			Proline	121 ± 18	206 ± 29 *
Catechin	1.9 ± 0.2	1.9 ± 0.2	Histidine	78 ± 11	536 ± 79 **
Epicatechin	0.4 ± 0.1	1.1 ± 0.1 ***	GABA	129 ± 17	202 ± 24 *
*Flavon-3-ols (µmol g^–1^ FW)*			Cysteine	85 ± 8	107 ± 12
Q-*O*-Rha-galactose	0.05 ± 0.01	0.05 ± 0.01	*^a^* *Hexose (mmol g^–1^ FW)*		
Q-*O*-Rha-glucose	0.12 ± 0.01	0.06 ± 0.01 ***	Glucose	7.73 ± 0.77	0.08 ± 0.01 **
Q-3-*O*-galactose	0.57 ± 0.07	2.25 ± 0.33 **	Fructose	5.85 ± 0.63	0.17 ± 0.03 **
Q-3-*O*-glucose	0.59 ± 0.06	1.85 ± 0.22 ***	Galactose	0.81 ± 0.15	0.04 ± 0.01 **
Q-*O*-arabinose	0.06 ± 0.01	0.05 ± 0.01	*^a^* *Disaccharides (µmol g^–1^ FW)*		
Q-*O*-xylose	0.30 ± 0.03	1.14 ± 0.10 ***	Sucrose	1.04 ± 0.15	0.24 ± 0.04 **
K-*O*-glucose-Rha-Rha	0.55 ± 0.07	0.25 ± 0.03 **	Trehalose	0.041 ± 0.005	0.024 ± 0.005
K-3-*O*-rutinose	0.30 ± 0.05	0.19 ± 0.02	*^a^* *Sugar alcohols (µmol g^–1^ FW)*		
K-3-*O*-galactose	0.78 ± 0.13	1.41 ± 0.06 ***	Sorbitol	0.09 ± 0.01	0.08 ± 0.01
K-3-*O*-glucose	0.33 ± 0.04	0.57 ± 0.05 **	Galactinol	0.07 ± 0.01	0.07 ± 0.01
K-*O*-arabinose	0.19 ± 0.02	0.29 ± 0.05	Mannitol	0.06 ± 0.01	0.07 ± 0.01
K-*O*-xylose	0.02 ± 0.01	0.06 ± 0.01 **	*^a^* *Trisaccharides (µmol g^–1^ FW)*		
*^a^* *Pentose (µmol g^–1^ FW)*			Maltotriose	0.023 ± 0.003	0.004 ± 0.001 *
Rhamnose	0.44 ± 0.05	0.12 ± 0.01 *	Raffinose	0.06 ± 0.01	0.07 ± 0.01

Values presented means ± SE (n = 3–6). Significant differences between WL and GL according to the *t*-test are indicated (* *p* < 0.05, ** *p* < 0.005, *** *p* < 0.001). CA, caffeic acid; GABA, γ-aminobutyric acid; K, kaempferol; *p*-HBA, *p*-hydroxybenzoic acid; *p*-CA, *p*-coumaric acid; PrcA, protocatechuic acid; Q, quercetin; Rha, rhamnose; SyA, syringic acid. ^a^ Data from Vidović et al. [1].

**Table 2:** Content of Fe, Cu, Ca, Mn, and Zn in GL and WL of *P. zonale* plants grown under optimal conditions.

Element	GL	WL
Fe, µg g^–1^_DW_	64.5 ± 6.2	102.5 ± 9.9 *
Cu, µg g^–1^_DW_	7.0 ± 0.6	11.1 ± 0.1 *
Ca, mg g^–1^_DW_	23.4 ± 2.7	10.8 ± 1.3 *
Mn, µg g^–1^_DW_	113.0 ± 4.3	243.0 ± 9.3 **
Zn, µg g^–1^_DW_	28.8 ± 2.1	50.4 ± 3.7 *

Values are given as means ± SE (n = 3). Significant differences between WL and GL, according to the *t*-test, are indicated (* *p* < 0.05, ** *p* < 0.005).

## Data Availability

Raw data from this article can be found in the Short Read Archive database at the NCBI database (https://www.ncbi.nlm.nih.gov/sra, accessed on 24 December 2022) under accession numbers SRX14189101, SRX14189100, SRX14189099, SRX14189098, SRX14189097, and SRX14189096 (three GL samples, and three WL samples) and Bioproject accession no. PRJNA807121 and Sample accession no. SAMN25962180—WL, and SAMN25962179—GL).

## Supplementary Materials

The following supporting information can be downloaded at: https://www.mdpi.com/article/10.3390/ijms24065288/s1.
