# Supplementary material for: Contrasting Metabolisms in Green and White Leaf Sectors of Variegated Pelargonium zonale—An Integrative Transcriptomic and Metabolomic Study
Source: Int J Mol Sci. 2023 Mar 9;24(6):5288. doi: 10.3390/ijms24065288 (PMC10048803; doi:10.3390/ijms24065288)
Supplement: Supplementary file 1 [file ijms-24-05288-s001.zip › Supplementary Figure S4.pdf]

A) Distribution of FPKM in three GL and three WL samples of variegated *P. zonale* leaves

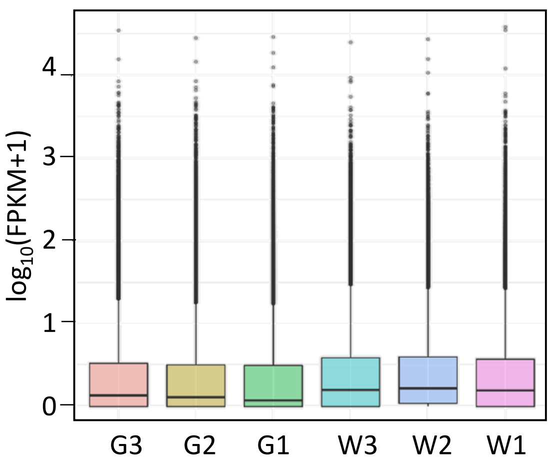

B) Heat map of the correlation coefficient between the samples

	W1	W2	W3	G1	G2	G3
W1	1.00	0.89	0.75	0.53	0.49	0.59
W2	0.89	1.00	0.93	0.73	0.70	0.79
W3	0.75	0.93	1.00	0.78	0.80	0.85
G1	0.53	0.73	0.78	1.00	0.88	0.88
G2	0.49	0.70	0.80	0.88	1.00	0.92
G3	0.59	0.79	0.85	0.88	0.92	1.00
