# Supplementary material for: Contrasting Metabolisms in Green and White Leaf Sectors of Variegated Pelargonium zonale—An Integrative Transcriptomic and Metabolomic Study
Source: Int J Mol Sci. 2023 Mar 9;24(6):5288. doi: 10.3390/ijms24065288 (PMC10048803; doi:10.3390/ijms24065288)
Supplement: Supplementary file 1 [file ijms-24-05288-s001.zip › Supplementary Table S1.pdf]

Supplementary Table S1. RNAseq data quality control and transcript length distribution.

Overview of data production quality

sample	Raw reads	Clean reads	Raw data(G)	Clean data(G)	Error rate (%)	Q20 (%)	Q30 (%)	GC content (%)
G1	39752969	38913224	11.9	11.7	0.04	98.27	94.72	47.85
G2	42418197	41680883	12.7	12.5	0.04	97.99	94.01	48.30
G3	39380222	38695746	11.8	11.6	0.04	98.04	94.15	48.31
W1	43889560	43352283	13.2	13.0	0.04	97.94	93.85	47.23
W2	38005994	37496402	11.4	11.2	0.04	98.07	94.17	46.84
W3	45754459	45337775	13.7	13.6	0.04	98.03	94.09	47.04

Overview of the length distribution of transcripts and unigenes

Type	Min Length	Mean Length	Median Length	Max Length	N50	N90	total nucleotides
Transcript	201	1218	819	37414	1873	542	170336510
Unigene	201	1220	821	37414	1874	543	170275938

Overview of the number of transcripts and unigenes in different length intervals

Transcript length interval	200-500 bp	500-1 kbp	1-2 kbp	> 2 kbp	Total
# of Transcripts	42738	37470	34641	24962	139811
# of Unigenes	42504	37468	34641	24962	139575
