# Supplementary material for: Contrasting Metabolisms in Green and White Leaf Sectors of Variegated Pelargonium zonale—An Integrative Transcriptomic and Metabolomic Study
Source: Int J Mol Sci. 2023 Mar 9;24(6):5288. doi: 10.3390/ijms24065288 (PMC10048803; doi:10.3390/ijms24065288)
Supplement: Supplementary file 1 [file ijms-24-05288-s001.zip › Supplementary Table S4.pdf]

Supplementary Table S4. Sequences of forward and reverse primers used for the detection of selected transcripts.

**Gene**	Forward primer	Reverse primer
POD17	CCGAATCCATTGTCCGGGAT	CCTCCTTAGCTTCGTCGACC
POD42	ACCCGGGTCTTGTCATGAAC	CTCTCTCTCCACAGCCTCCT
MDAR	GAGGTGGAAATTCGGCTGGA	ACAAGTGTGGAAACCAGGCA
APX	CTTCCACCCCTAGGCTCTCT	CTTCAGCTGGCTAGGATCCG
DFR	CAAGCGGCTTGGAAGTATGC	GGCTTTGGGGTGCTCATACA
Actin	AGCTGAGAGATTCCGTTGCC	CCTTGCTCATCCTGTCAGCA
CHS1	GCCTTCGAGCCTTTGGGTAT	CAGCCCATCCTCACACGATT

POD, class III peroxidase; MDAR, monodehydroascorbate reductase; APX, ascorbate peroxidase; DFR, dihydroflavonol 4-reductase; CHS, chalcone synthase.
