# Supplementary figures and images for: Contrasting Metabolisms in Green and White Leaf Sectors of Variegated Pelargonium zonale—An Integrative Transcriptomic and Metabolomic Study
Source: Int J Mol Sci. 2023 Mar 9;24(6):5288. doi: 10.3390/ijms24065288 (PMC10048803; doi:10.3390/ijms24065288)

A) E-value distribution

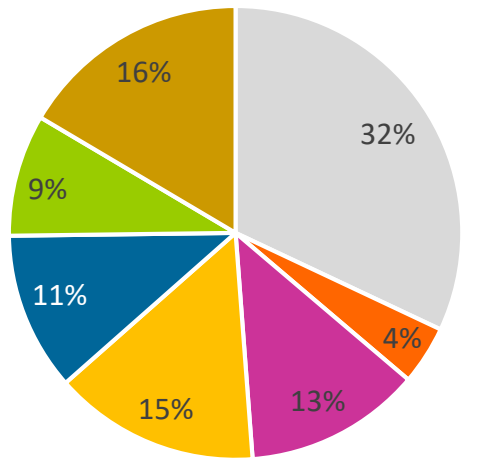

B) Similarity distribution

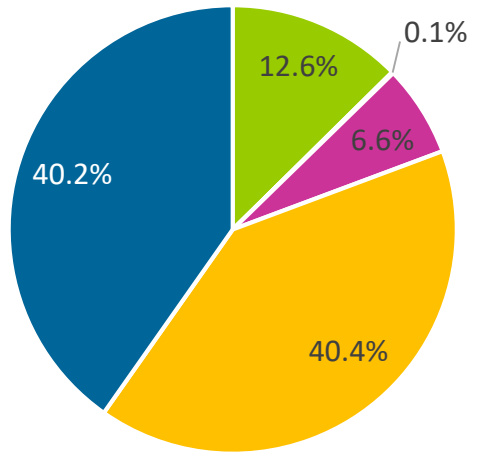

C) Species distribution

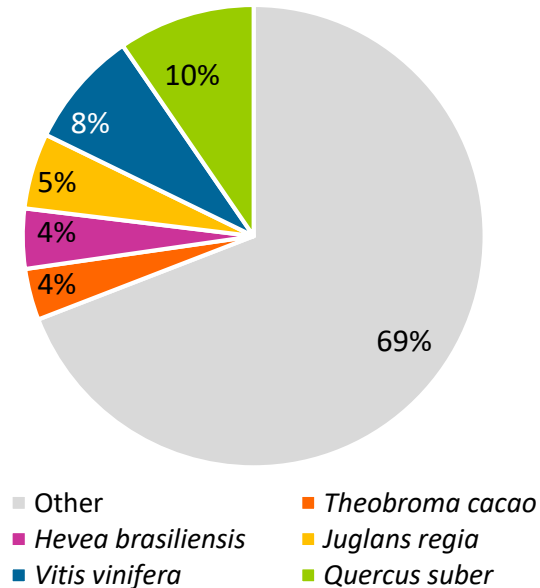

Supplement: Supplementary file 1 [file ijms-24-05288-s001.zip › Supplementary Figure S1.pdf]

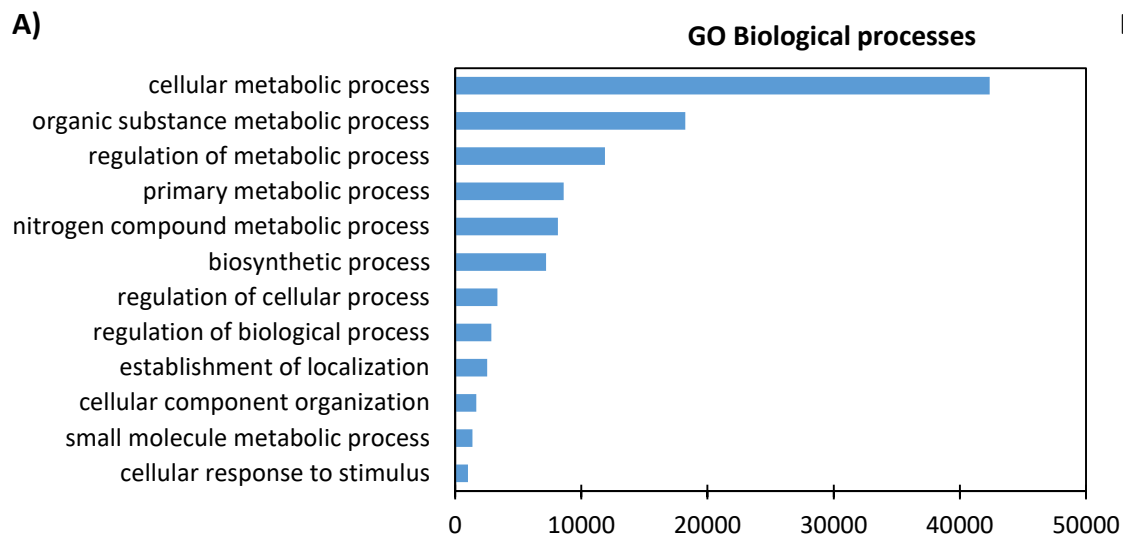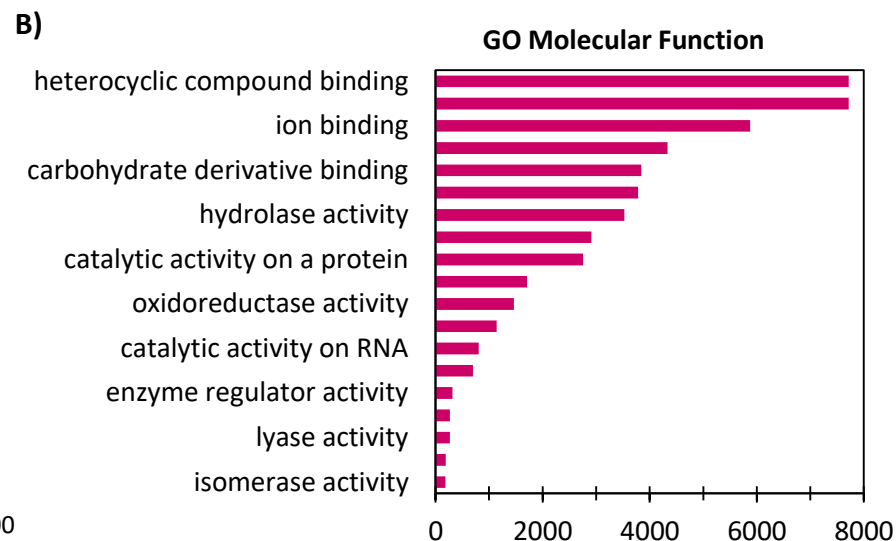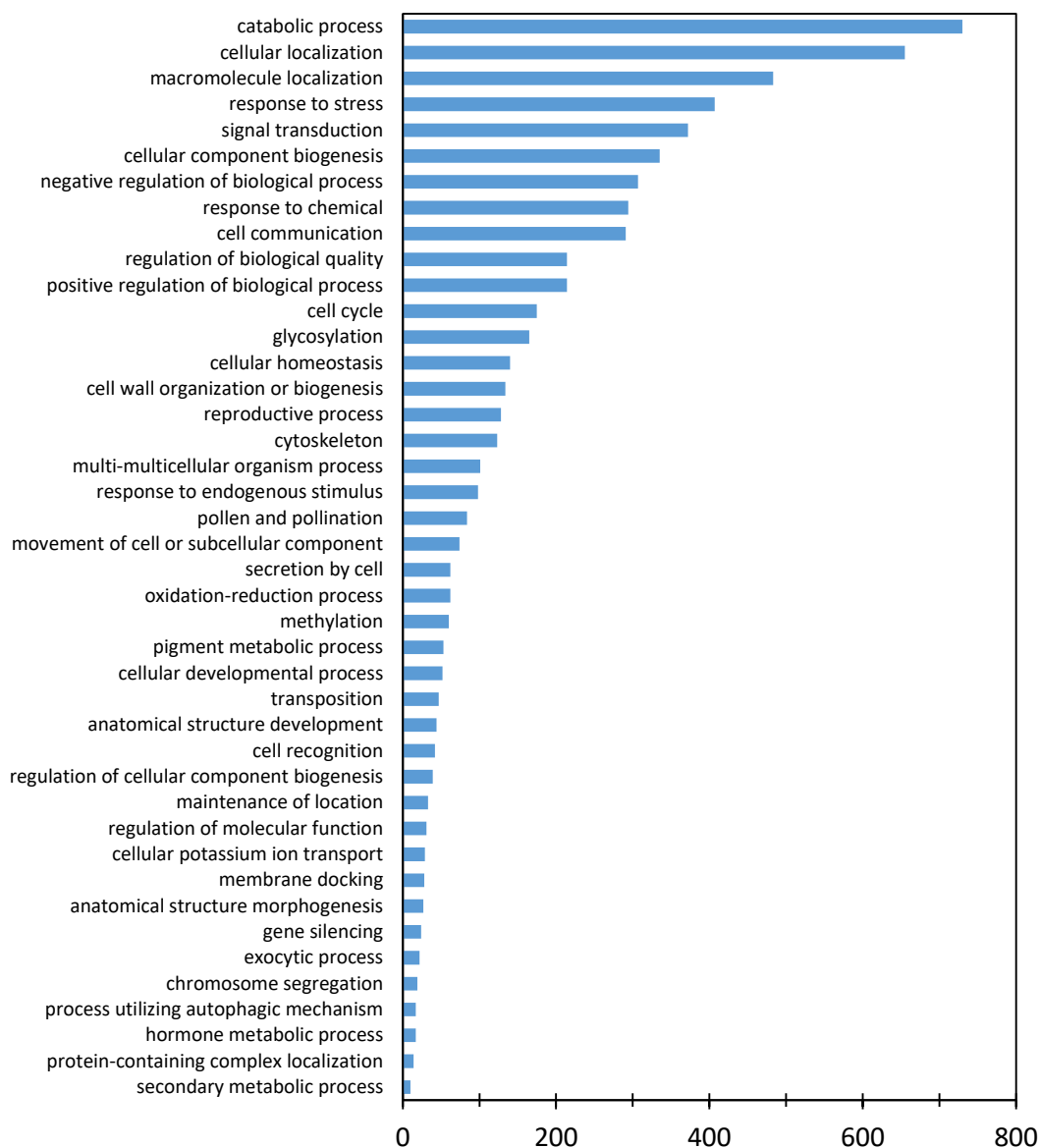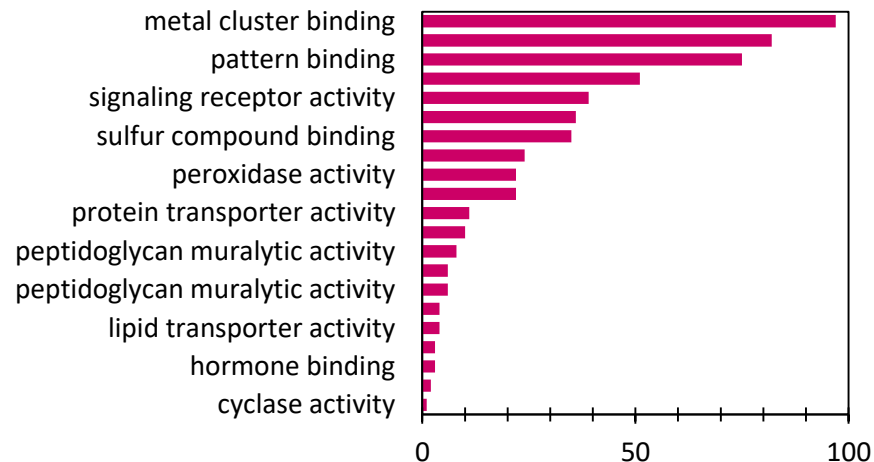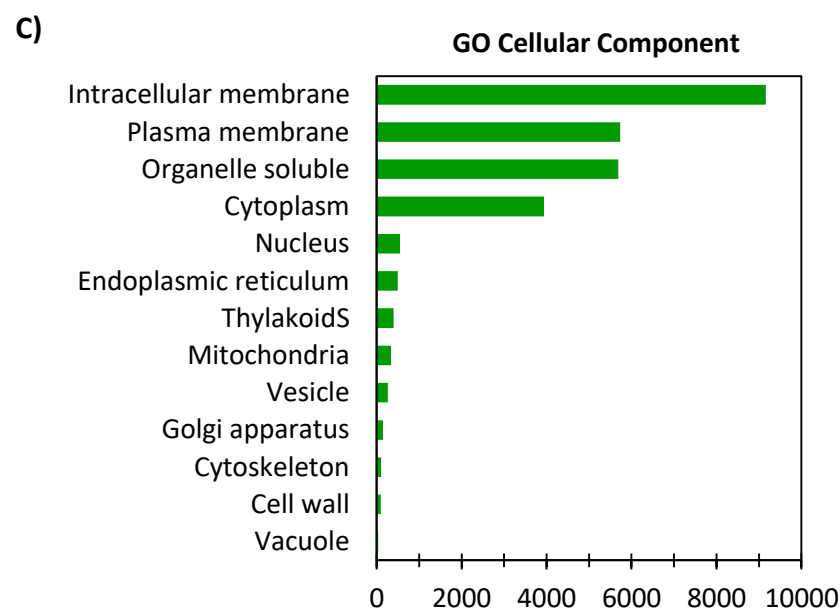

Supplement: Supplementary file 1 [file ijms-24-05288-s001.zip › Supplementary Figure S2.pdf]

## KOG Annotation

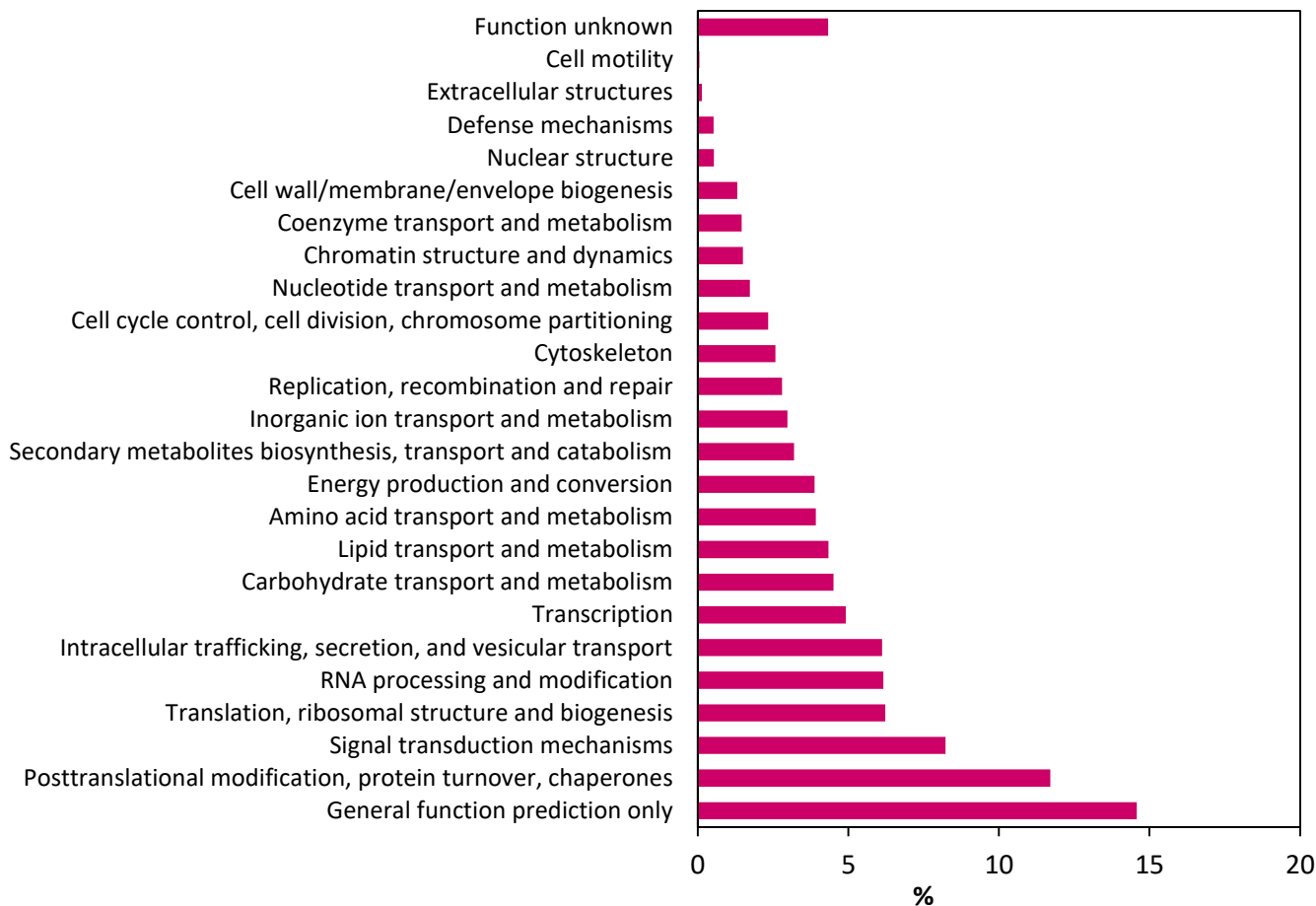

Supplement: Supplementary file 1 [file ijms-24-05288-s001.zip › Supplementary Figure S3.pdf]

Correlation between transcriptome data and real time PCR results of six selected genes.

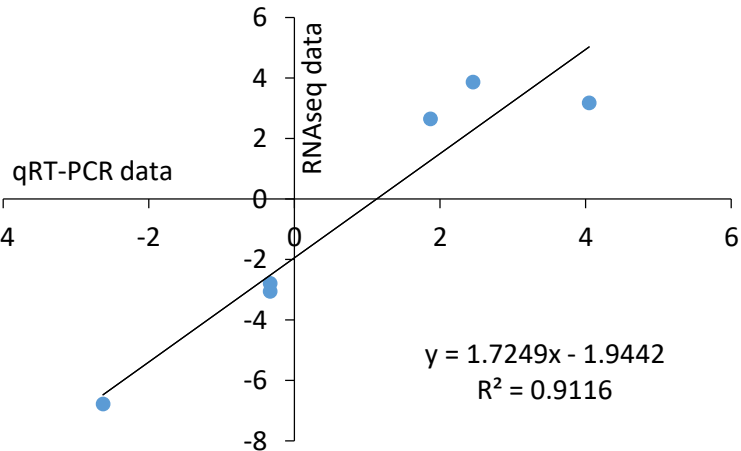

Supplement: Supplementary file 1 [file ijms-24-05288-s001.zip › Supplementary Figure S6.pdf]
