# Supplementary material for: Contrasting Metabolisms in Green and White Leaf Sectors of Variegated Pelargonium zonale—An Integrative Transcriptomic and Metabolomic Study
Source: Int J Mol Sci. 2023 Mar 9;24(6):5288. doi: 10.3390/ijms24065288 (PMC10048803; doi:10.3390/ijms24065288)

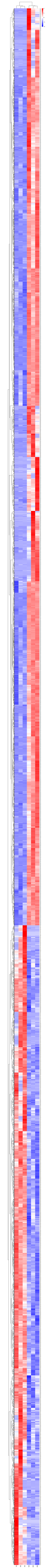

Supplement: Supplementary file 1 [file Supplementary Figure S5.pdf › Supplementary Figure S5.pdf]
